# Long-Term Resilience of Late Holocene Coastal Subsistence System in Southeastern South America

**DOI:** 10.1371/journal.pone.0093854

**Published:** 2014-04-09

**Authors:** André Carlo Colonese, Matthew Collins, Alexandre Lucquin, Michael Eustace, Y. Hancock, Raquel de Almeida Rocha Ponzoni, Alice Mora, Colin Smith, Paulo DeBlasis, Levy Figuti, Veronica Wesolowski, Claudia Regina Plens, Sabine Eggers, Deisi Scunderlick Eloy de Farias, Andy Gledhill, Oliver Edward Craig

**Affiliations:** 1 BioArCh, Department of Archaeology, University of York, York, United Kingdom; 2 Department of Physics, University of York, York, United Kingdom; 3 York Centre for Complex Systems Analysis (YCCSA), University of York, York, United Kingdom; 4 Department of Biology, University of York, York, United Kingdom; 5 Department of Archaeology, Environment and Community Planning, La Trobe University, Melbourne, Australia; 6 Museu de Arqueologia e Etnologia (MAE), Universidade de São Paulo (USP), São Paulo, Brazil; 7 Laboratório de Estudos Arqueológicos (LEA), Departamento de História, Universidade Federal de São Paulo (UNIFESP), São Paulo, Brazil; 8 Laboratório de Antropologia Biológica, Departamento de Genética e Biologia Evolutiva, Instituto de Biociências, Universidade de São Paulo (USP), São Paulo, Brazil; 9 Grupep, Universidade do Sul de Santa Catarina (UNISUL), Tubarão, Brazil; 10 Division of Archaeology, Geography and Environmental Sciences, University of Bradford, Bradford, United Kingdom; University of Oxford, United Kingdom

## Abstract

Isotopic and molecular analysis on human, fauna and pottery remains can provide valuable new insights into the diets and subsistence practices of prehistoric populations. These are crucial to elucidate the resilience of social-ecological systems to cultural and environmental change. Bulk collagen carbon and nitrogen isotopic analysis of 82 human individuals from mid to late Holocene Brazilian archaeological sites (∼6,700 to ∼1,000 cal BP) reveal an adequate protein incorporation and, on the coast, the continuation in subsistence strategies based on the exploitation of aquatic resources despite the introduction of pottery and domesticated plant foods. These results are supported by carbon isotope analysis of single amino acid extracted from bone collagen. Chemical and isotopic analysis also shows that pottery technology was used to process marine foods and therefore assimilated into the existing subsistence strategy. Our multidisciplinary results demonstrate the resilient character of the coastal economy to cultural change during the late Holocene in southern Brazil.

## Introduction

The Brazilian coast encompasses a wide range of tropical and sub-tropical ecosystems that have sustained human populations from the middle Holocene to the present day. The large shell mounds, or sambaquis, are a distinctive feature of this coastline, testament to large-scale exploitation of marine resources, from ∼8,000 to ∼1,000 calibrated years before present (cal BP). In southern Brazil some sambaquis reached more than 35 m high and contained hundreds of burials, post holes and faunal remains testifying the development of a complex social panorama [1]. The exploitation of aquatic (mostly marine) resources was an important subsistence activity at these sites [2] and must have drawn people to the coast. However indirect evidence reveals that the contribution of plants also appears to be important [3]–[8]. Sambaquis containing freshwater and land snail shells are also found along the courses of rivers and their distribution penetrates some distances inland. These “Riverine sambaquis” are the same age or even older than their coastal analogues (∼10,000 to ∼1,000 cal BP) [9] and occasionally finds of marine fauna at these riverine sites suggest some connection to the coast [10], [11], [12].

A dramatic change is seen in the archaeological record at ∼1,500 cal BP with the abrupt cessation of large shell mound formation [13]. At this time it is thought that new populations from the southern highlands (known as the Taquara/Itararé tradition) [14] expanded to the coastal lowlands [15], likely driven by rapid population growth, increasing of social interaction and intensification in food production, involving maize and exploitation of pine forest (*Araucaria angustifolia*) [16]. The appearance of Taquara/Itararé pottery along the southern coast of Brazil therefore may mark a key turning point in exploitation of rich coastal ecotones, as prehistoric groups gained the knowledge and technology to develop new economic practices. However the extent to which the transmission (or imposition) of this new subsistence system transformed the indigenous coastal economy, and its capacity to adjust, persist and maintain its fundamental properties, is still a matter of debate [17].

While there is some evidence for consumption of new cultigens like maize by Taquara/Itararé groups [7] and increased consumption of terrestrial resources [18], marine fauna continue to be found at high abundance [19]. Similarly, while pottery is often assumed to be associated with the processing of new produced and foraged foods, with parallels in the southern Brazilian Highlands [16], there is no direct evidence of what it was used for. Our understanding of the diet and subsistence economy of the prehistoric inhabitants of coastal Brazil has been largely limited to traditional archaeological information, based on faunal, botanical and artefactual remains. In particular, the contribution of marine and terrestrial foods to the diet of both pre-ceramic and ceramic coastal populations of this region still remains largely unknown [11], [20], [21] and only few studies have considered the use of ceramics during this period [22], [23]. As a result, the impact of new economic and technological strategies on coastal adapted hunter-gatherers societies is not yet understood.

Here we report the results of an integrated study into the dietary variability of coastal and inland sambaqui populations. We analysed the stable carbon and nitrogen isotope composition of human bone collagen, a technique widely used to reconstruct paleodiets, and particularly for distinguishing marine versus terrestrial diets [24], [25]. We determined the stable carbon isotope signature of individual amino acids from bone collagen to identify different macronutrient constituents of diet [26]–[28]. We assessed the potential of bone mineral for isotope analysis in order to provide information on whole diet [24]. Finally we considered additional information regarding the diet of ‘incoming’ ceramic producing groups through the analysis of the organic contents of their pottery [29]–[31]. Hansel et al. [22] and Hansel and Schmitz [23] have already shown the potential for retrieving lipids from pottery in coastal Brazil, but here we report the first compound-specific isotopic analysis of these artefacts.

### Archaeological Setting

**Figure 1:**
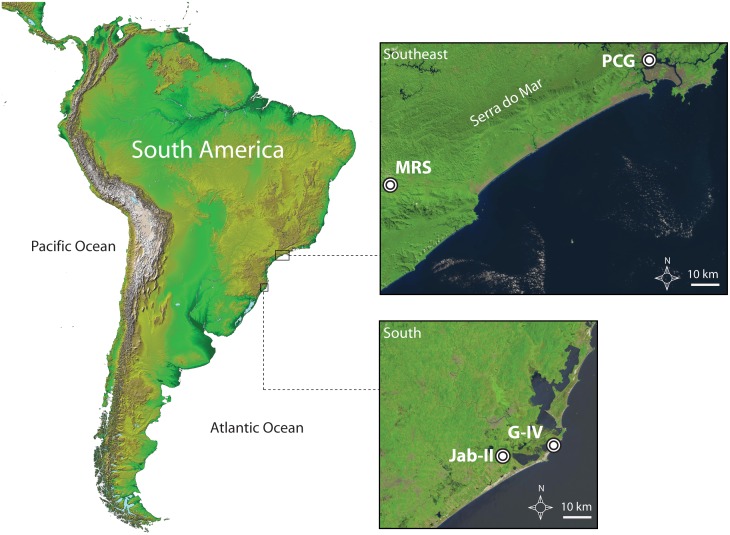
Geographic position of the analysed archaeological records. Location of the study area showing the archaeological sites from southeast (PCG, MRS) and south (Jab-II, G-IV) regions of Brazil. Satellite imagery from USGS (EarthExplorer) and NASA (Shuttle Radar Topography Mission).

The archaeological records include four middle and late Holocene sites located in southeast (São Paulo) and southern (Santa Catarina) regions of Brazil (Fig. 1). These consist of one inland riverine site (Moraes) and two coastal sambaquis (Jabuticabeira II, Piaçaguera), along with a recently excavated ceramic coastal site (Galheta IV), dated to the time of the expansion of the highland groups to the coast. The sambaqui sites dated to the preceramic period show a wide range, but well-established chronology (∼6,700 to ∼1,700 cal BP) and offer a unique opportunity to elucidate dietary variability between coastal and inland mound builders. They also offer a valuable isotopic baseline for assessing changes in subsistence strategies associated with the spread of pottery technology (∼1,500 cal BP). Preliminary stable isotope studies have already been carried out on human remains from some of these sites (Moraes, Jabuticabeira II) [11], [21] as well as other coastal sites in southern Brazil [20], [32]. Here we undertake the analysis of 106 human remains with the aim of greatly expanding the dietary isotope record for this region.

Moraes (MRS) is situated in the Ribera do Iguape Valley, at ∼35 km from the São Paulo coast. The site forms a mound with a conspicuous concentration of land snail shells dated between 6,775–6,499 and 5,289–4,887 cal BP [33]. The mound was used mainly for funerary purposes [34] and 55 human individuals were recorded in the deposit [34], [35], along with a consistent amount of faunal remains mostly represented by terrestrial mammals, followed by amphibians, birds, and only few reptiles and fish bones [10], [11].

Piaçaguera (PCG) is located in the south-east coastal plain of São Paulo state, at ∼12 km from the present day shoreline. PCG similarly provided a stratigraphic succession containing more than 80 human individuals, artefacts and faunal remains dated to 5,894–5,326 and 5,887–5,314 cal BP [35], [36]. Fish and molluscs dominate the assemblage, followed by some crustaceans, a few terrestrial mammals and carbonized seeds [35]–[38].

Jabuticabeira II (Jab-II), at ∼7 km from the present day shoreline at Santa Catarina, is one of the best-studied sambaqui sites of Brazil [21], [39]. The stratigraphic sequence is composed of alternating layers of marine shells and fish bones [21]. Numerous human burials and the absence of compelling domestic activities point to a ceremonial function of the site from 3,137–2,794 to 1,860–1,524 cal BP [13], [40].

Finally, Galheta IV (G-IV) is a funerary site overlooking the Atlantic Sea, at ∼20 km north of Jab-II, in Santa Catarina [41]. The site yielded 8 adult human burials, some of them partially cremated, associated with abundant marine faunal remains (fish, sea mammals, seabirds) and artefacts, including potsherds of Taquara/Itararé tradition. G-IV was ^14^C dated between 1,304–1,140 and 913–739 cal BP, and it dates to the expansion of inland ceramic producers (southern Jê speakers) to the coast.

## Material and Methods

### Human and Faunal Remains: Sampling Procedure and Ethical Statement

A total of 106 human individuals (different age and sex) from MRS, PCG, Jab-II and G-IV were sampled for isotopic analysis. In order to build a faunal isotopic reference for the region, 36 animal bone remains (terrestrial mammals, sea mammals, birds and fish) from these four sites were also selected for isotopic analysis (Tab. 1). Human samples were obtained almost entirely from the ribs, whereas fauna samples were from a range of different skeletal elements. All necessary permits were obtained for the described study, which complied with all relevant regulations of the Instituto do Patrimônio Histórico e Artístico Nacional – IPHAN (protocols n° 01506.00407/2012-14, 01506.001516/2006-47 and 01510.000047/2003-37). Archaeological materials used in this study are stored at the University of York (UK), Biology S-Block.

**Table 1:** Bone collagen δ^13^C and δ^15^N values of faunal remains.

Site	Taxon	Vernacular name	δ^13^C%	δ^15^N%	%C	%N	C:N	Col wt%	Source
MRS	*Tayassu* sp.	Peccary	–21.0	+8.6	27.0	9.6	3.3	0.5	This study
MRS	*Tayassu* sp.	Peccary	–21.3	+6.7	37.4	13.4	3.3	1.4	This study
MRS	*Tayassu* sp.	Peccary	–23.5	+9.2	35.6	12.7	3.3	1.5	This study
MRS	*Tayassu* sp.	Peccary	–22.4	+8.7	40.5	14.9	3.2	1.5	This study
MRS	*Mazama* sp.	Brocket	–23.0	+9.6	31.6	11.2	3.3	0.6	This study
MRS	*Mazama* sp.	Brocket	–24.6	+8.4	41.0	14.7	3.2	1.5	This study
MRS	*Cuniculus paca*	Lowland paca	–20.8	+7.9	42.5	14.4	3.4	1.2	This study
MRS	*Cuniculus paca*	Lowland paca	–20.6	+8.7	41.7	14.8	3.3	1.0	This study
MRS	*Alouatta* sp.	Howler monkeys	–21.5	+7.9	26.0	9.3	3.3	3.3	This study
MRS	*Alouatta* sp.	Howler monkeys	–22.5	+8.6	43.0	15.5	3.2	1.4	This study
MRS	*Tayassu* sp.	Peccary	–22.3	+6.5	-	-	-	-	[11]
MRS	*Alouatta* sp.	Howler monkeys	–21.6	+6.3	-	-	-	-	[11]
PCG	Selachimorpha	Shark	–11.8	+15.2	29.2	9.8	3.5	3.0	This study
PCG	*Euphractus sexcinctus*	Six-banded armadillo	–20.3	+13.3	40.5	13.7	3.4	1.9	This study
PCG	*Alouatta* sp.	Howler monkeys	–22.5	+6.9	56.4	19.8	3.3	2.0	This study
Jab-II	*Trichiurus lepturus*	Hairtail	–11.1	+12.8	41.1	14.8	3.2	3.5	This study
Jab-II	*Lobotes surinamensis*	Tripletail	–10.5	+14.5	41.9	14.6	3.4	2.3	This study
Jab-II	*Pogonias cromis*	Blackdrum	–9.8	+12.6	41.2	14.3	3.4	3.4	This study
Jab-II	Ariidae	Sea catfishes	–9.2	+15.5	42.1	15.1	3.3	2.7	This study
Jab-II	Pomacanthidae	Angelfishes	–10.8	+13.4	41.5	14.4	3.4	1.6	This study
Jab-II	Cetacea	Undeter	–10.8	+16.1	39.5	13.8	3.3	2.2	This study
Jab-II	Aves	unknown bird	–19.5	+7.2	42.3	15.0	3.3	4.8	This study
G-IV	Aves	unknown seabird	–12.5	+17.4	43.3	15.7	3.2	6.6	This study
G-IV	Aves	unknown seabird	–9.8	+18.9	44.3	16.0	3.2	6.2	This study
G-IV	Aves	unknown seabird	–13.6	+17.9	42.7	15.5	3.2	4.6	This study
S Brazil	*Arctocephalus tropicalis*	Subantarctic fur seal	–11.0	+16.0	-	-	-	-	[20]
S Brazil	*Arctocephalus australis*	S American fur seal	–11.4	+16.4	-	-	-	-	[20]
S Brazil	*Eubalaena australis*	Southern right whale	–15.2	+6.9	-	-	-	-	[20]
S Brazil	*Spheniscus magellanicus*	Magellanic penguin	–11.2	+14.5	-	-	-	-	[20]
S Brazil	Selachimorpha	Shark	–9.5	+16.0	-	-	-	-	[20]
S Brazil*	*Macrodon ancylodon*	King weakfish	–11.8	+13.0	60.0	22.4	3.1	11.0	This study
S Brazil*	*Epinephelus marginatus*	Dusky grouper	–11.1	+16.0	44.1	16.9	3.0	19.4	This study
S Brazil*	*Micropogonias furnieri*	whitemouth croaker	–11.3	+13.9	43.5	16.4	3.1	13.5	This study
S Brazil*	*Peprilus paru*	American harvestfish	–15.0	+13.0	49.9	14.7	4.0	11.1	This study
S Brazil*	Mugilidae	Mullets	–12.5	+11.5	41.5	15.5	3.1	12.8	This study
S Brazil*	*Cynoscion acoupa*	Acoupa weakfish	–9.0	+11.3	43.3	16.4	3.1	10.7	This study
S Brazil*	*Urophycis* sp.	Brazilian codling	–10.9	+14.2	41.0	15.6	3.1	13.0	This study
S Brazil*	*Coryphaena* sp.	Common dolphinfish	–13.3	+9.2	41.9	15.8	3.1	16.1	This study
S Brazil*	*Pomatomus saltatrix*	Blue fish	–11.9	+15.3	55.7	20.8	3.1	7.8	This study
S Brazil*	*Xiphias gladius*	Swordfish	–12.1	+9.9	70.9	26.2	3.2	11.5	This study

Bone collagen δ^13^C and δ^15^N values are also reported from previous studies [20], [11] and for modern (*) fish from the south Brazilian coast, after correction for the decrease of δ^13^C values of atmospheric CO_2_.

Isotopic analyses were also conducted on modern fish (n = 10) caught using traditional fishing techniques and acquired at the central market of Florianópolis (Santa Catarina, S. Brazil). The use of modern fish specimens was carried out in strict accordance with the recommendations of the Brazilian Institute of Environment and Renewable Natural Resources – IBAMA. No *In Vivo* experiment was developed, thus ARRIVE guidelines (Animal Research: Reporting *In Vivo* Experiments) for this study is not applicable. The transport of modern specimens to the University of York was approved under the Convention on International Trade in Endangered Species of Wild Fauna and Flora – CITES-IBAMA (protocol n° 113508). Modern fish collagen δ^13^C were corrected (+1.14%) for the global decrease of atmospheric δ^13^C values [42]. We also incorporated δ^13^C and δ^15^N values from Plens [11] and De Masi [20], who report collagen isotopic composition of archaeological faunal remains from MRS and other coastal sites of southern Brazil.

### Assessing Bone Collagen and Mineral Preservation

Bulk collagen δ^13^C and δ^15^N derive primarily from dietary protein, although macronutrients (carbohydrates, lipids) may variably contribute to collagen carbon, particularly for the non-essential amino acids in collagen [43]–[45]. The δ^13^C of bone apatite instead reflects the total dietary pool of carbon ingested [46]. Therefore the combination of collagen and apatite δ^13^C has been shown to give information on main energy and protein consumed several years prior to death [47], [48]. In archaeological contexts, however, the burial environment may impact the physical and chemical composition of bones in different ways [49], particularly through loss of collagen and alteration of bio-apatite and the stable isotope signature of this fraction [50].

Assessment of collagen preservation in both human and faunal remains was carried out following the criteria proposed by van Klinken [51]. In addition, Raman spectroscopy studies were performed on randomly selected human bone samples from MRS (n = 9), Jab-II (n = 9), G-IV (n = 4) and PCG (n = 10) to assess diagenetic change to the mineral fraction, with modern lamb bone being used as a control [52]. To optimise the quality of the Raman spectra, the samples of bone were flattened and smoothed by gentle rubbing with fine-grade diamond paper. The Raman spectra were collected from the samples using an HORIBA XploRa instrument at 532 nm laser wavelength and under x100 magnification in confocal mode (NA = 0.9, with 2400 g mm-1 grating). Five spectra were collected from each bone specimen using 1s laser exposure at ∼3.5 mW power at the sample, with each measurement averaged over 40 spectral acquisitions. The spectra were collected over 4 spectral windows to achieve a total spectral range of 200–3200 cm^−1^. The software package IGOR Pro 6.32 was used to average, baseline correct, and analyse the Raman spectra using Gaussian peak-fitting procedures, with the ν_1_ carbonate peak (at ∼1070 cm^−1^) de-convoluted according to published protocols [53].

### Collagen Extraction and Isotope Analysis

Collagen preparation follows the protocol described in Craig et al. [54]. Before isotopic analysis, lipids were removed from modern fish bones with dichlormethane:methanol (2∶1, x3). Between 300 and 500 mg of cleaned human and animal bones were used for collagen extraction. Samples were agitated in 8 ml of 0.6 M hydrochloric acid at 4°C to demineralize. Once demineralization had occurred the samples were removed from the acid and washed with ultrapure water three times. The samples were gelatinised in pH 3 hydrochloric acid and maintained for forty-eight hours at 80°C. The gelatinised samples were then ultrafiltered and a >30 kDa fraction was lyophilised. Duplicates (1 mg) were measured using a continuous flow isotope ratio mass spectrometry Thermo Finnigan Delta Plus XL in the Department of Archaeological Sciences of the University of Bradford (UK), to determine the δ^13^C and δ^15^N values. The results are reported using the delta scale in % relative to internationally accepted standards, V-PDB and AIR respectively. Analytical error, calculated from repeated measurements of each sample and measurements of the bovine control from multiple extracts, was <0.2% (1σ).

### Analysis of Individual Amino Acids in Collagen

Stable carbon isotope analyses were performed on collagen amino acids isolated from randomly selected coastal individuals from Jab-II (n = 10) and G-IV (n = 7). Approximately 1 mg of collagen was hydrolysed under vacuum in amino acid free 6 M hydrochloric acid (1 ml) at 110°C for 24 hours. After hydrolysis the samples were dried in a rotary vacuum concentrator and stored at –20°C until analysis. Prior to isotopic analysis, the samples were redissolved under sonication in MilliQ water with the addition of an internal standard (2-amino-isobutyric acid).

Instrumental analysis was carried out using Thermo Scientific Liquid chromatography isotope ratio mass spectrometry (LC-IRMS) system consisting of an Accela 600 pump connected to a Thermo Scientific LC Isolink and a Delta V Plus Isotope Ratio Mass Spectrometer housed at the La Trobe Institute for Molecular Sciences (LIMS, La Trobe University, Melbourne, Australia). LC-IRMS analysis of single amino acid fractions of collagen hydrolysates were carried out using a three phase LC/IMRS method similar to that described in Smith et al. [55], with the exception that a Primesep A (SCIELC) column (2.1×250 mm, 100 Å, 5 μm) was used. This is a narrower column to that used by Smith et al. [55] and thus lower LC flow rates were used. Conditioning runs were made at 110 μLmin^−1^ using phases ratios of 85B:15C–95B:5C, analytical runs were made using flow rates of 60–80 μLmin^−1^ and oxidation reagent flow rates were set at 35 μLmin^−1^ each. Approximately 10 μg of amino acid hydrolysate (on column) was used for each run. Amino acid peak δ^13^C values were measured against CO_2_ gas pulses throughout the run (δ^13^C_VPDB_ = –2.8%) calibrated against international standard USGS-40 L-Glutamic Acid (δ^13^C_VPDB-LSVEC_ = –26.4±0.04%). In house standard runs were made during the sample runs to monitor measurement quality.

### Organic Residues Analysis

Molecular and isotopic analysis of organic residues absorbed into porous vessels or preserved in surface deposits offer valuable information concerning pottery use [29], [30], [54], [56]–[58]. Lipids were extracted (and methylated in one-step) from 14 potsherds according to protocols reported in Craig et al. [31], [59]. Briefly, after cleaning the surface, methanol (4 ml) was added to powdered ceramic samples (70 to 240 mg) and the mixture was sonicated for 15 min and then acidified with concentrated sulphuric acid (800 ml). The acidified suspension was heated in sealed tubes for 4 h at 70°C and then cooled, and lipids were extracted with n-hexane (2 ml×3). The extract was dried under a gentle flux of nitrogen and internal standard (n-hexatriacontane) was added before the direct analysis by gas chromatography/mass spectrometry (GCMS) at the University of York (UK).

Stable isotopic analysis of n-hexadecanoic (C_16∶0_) and n-octadecanoic (C_18∶0_) acids from 11 extracted lipid samples were performed using a gas chromatograph (GC) coupled to a combustion isotope ratio mass spectrometry (GC-C-IRMS) at the University of Liverpool (UK) following the protocol reported in Craig et al. [59]. Instrument precision on repeated measurements was 0.2% (s.e.m.).

Charred residues of food were preserved in the internal part of 6 ceramic potsherds. Samples (3–7 mg) were removed and subsamples (1 mg) selected for carbon and nitrogen isotopic analysis at the University of Bradford by using the same IRMS procedure as for bone collagen [56].

### Statistical Analysis

The proportional contribution of different food sources to human diet (based on stable isotope values) was estimated using a Bayesian mixing model in SIAR V4 (Stable Isotope Analysis in R) [60]. The bulk δ^13^C and δ^15^N values of faunal remains (Tab. 1) was used in the model calculation after adding trophic enrichments of 1% and 4% for carbon and nitrogen respectively [61]. Bulk δ^13^C and δ^15^N were also analysed using the parametric One-Way Anova test in the software PAST 2.13 [62], after checking for normal distribution (Shapiro-Wilk) and using a statistical significance probability threshold of α = 0.05. Available radiocarbon ages were calibrated with OxCal 4.2, using the Southern Hemisphere curve SHCal04 [63], [64].

## Results

### Bone Apatite and Collagen Preservation

**Figure 2:**
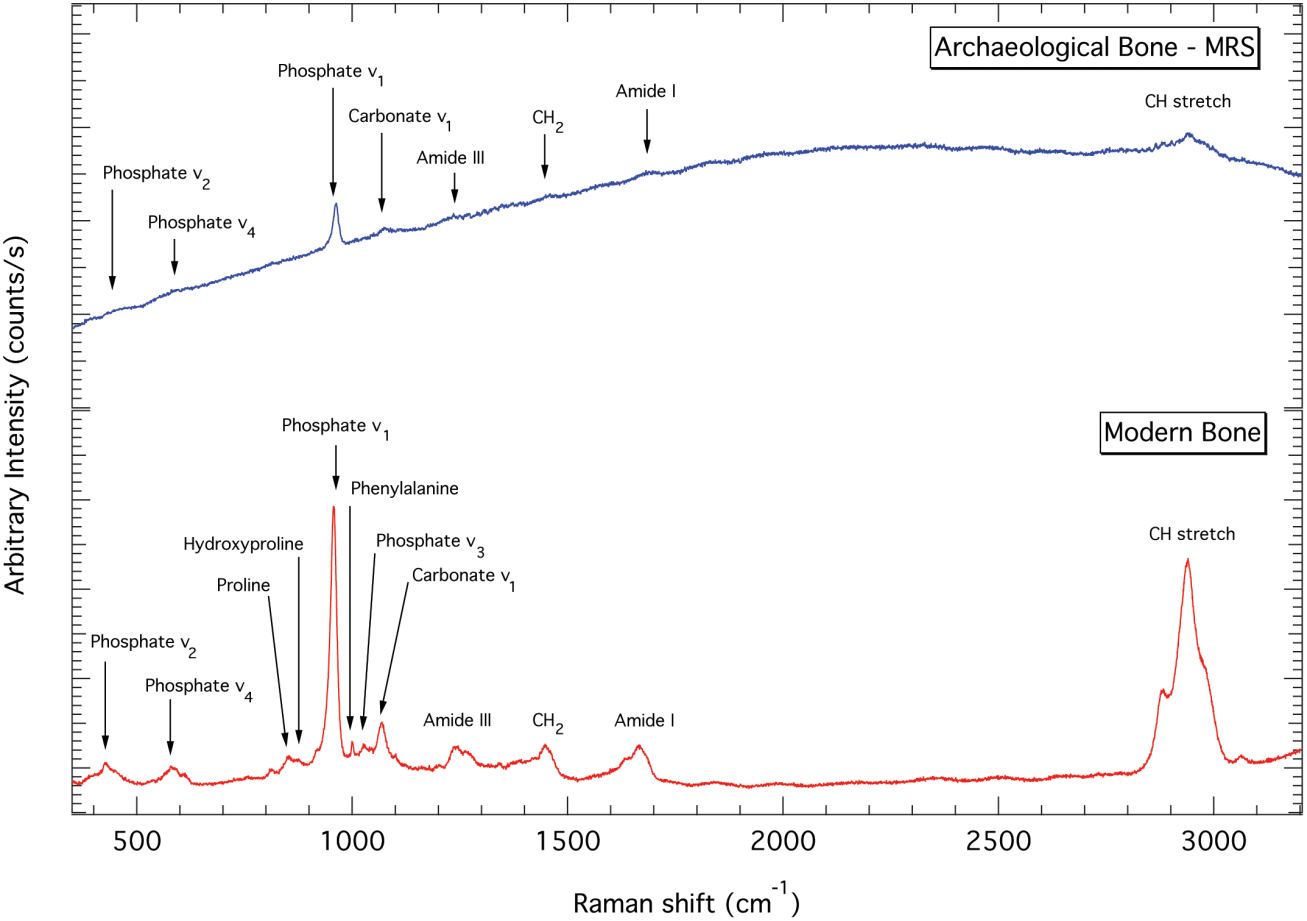
Bone diagenesis. Examples of Raman spectra of an archaeological bone spectrum (MRS) and a modern lamb bone without baseline correction showing the Raman band assignments of the key peaks. All peaks are identified as per the literature [68] and [112], with results being comparable to those described in Edwards et al. [69].

Raman analysis revealed substantial alteration to the mineral phase of all of the archaeological samples that were analysed (Tab. S1, Fig. 2). Substitution of non-biogenic carbonate for biogenic phosphate, or reduction in biogenic phosphate, is indicated by the increased carbonate ν_1_ (C) to phosphate ν_1_ (P) intensity ratios in archaeological bones compared to the modern control [65], [66]. An increase in crystallinity due to mineral alteration in the archaeological specimens is expected [49], [67] and confirmed in our Raman data by a decrease in the full width at half maximum (FWHM) of the ν_1_ P band (at ∼957 cm^−1^) relative to the modern bone sample [65], [66], [68]. Increasing crystallinity in diagenetically altered bone has been linked to the loss of collagen [49], which is further corroborated in our data by reduced CH/P ratios compared to the modern bone sample. Here, CH refers to the collagen band (i.e. CH stretching) at ∼2933 cm^−1^. The CH/P trend is also in agreement with the Raman spectroscopy results of Edwards et al. [69], which demonstrated reduced intensity in the collagen modes in human bones collected at JAB-II. Finally, the large fluorescent background in the archaeological samples (Fig. 2) is attributed to spectral emission from luminescent ions incorporated into the bone lattice due to diagenetic alteration [65].

Despite widespread alteration to the bone mineral phase, most of human burials (82 out of 106) matched the criteria for adequate collagen preservation [51]. Collagen yield and C:N ratios individuals from MRS (n = 15), PCG (n = 11), Jab-II (n = 47) and G-IV (n = 6) range from 0.9 to 7.7 wt% and from 3.2 to 3.5 respectively (Tab. SI2). Three individuals from PCG and one from G-IV contain <1% collagen but still had acceptable C:N ratios and their δ^13^C and δ^15^N values are coherent with the other humans. Similarly, most of the animal samples from the study sites (25 out of 36) had adequate (>1%) collagen yields (Tab. 1). Of these, one peccary (*Tayassu* sp.) and one brocket (*Mazama* sp.) from MRS show collagen yield of 0.5 and 0.6 wt% respectively, but again the C:N matches the criteria for unaltered collagen. In summary, whilst the bone mineral fraction is unlikely to preserve a vital isotopic signal, the collagen yield and C:N composition suggests acceptable collagen preservation for the majority of the samples [51], [70].

### Bulk Collagen Stable Isotope Analysis

Terrestrial fauna comprehensively show average δ^13^C and δ^15^N of −22.0±1.2% and +8.4±1.7% respectively (Tab. 1). Some variability is observed in carbon isotopes at MRS and may be a result of hunting in different environments. The aquatic fauna, modern and archaeological fish, exhibits average δ^13^C and δ^15^N of −11.1±1.5% and +13.5±1.8% respectively. Sea mammals show average δ^13^C and δ^15^N values of −12.3±1.9% and +12.9±4.5%. Seabirds show average δ^13^C of −13.3±3.7% and the highest average δ^15^N value among faunal remains, +15.2±4.8%, pointing to the consumption of higher trophic levels marine resources.

**Figure 3:**
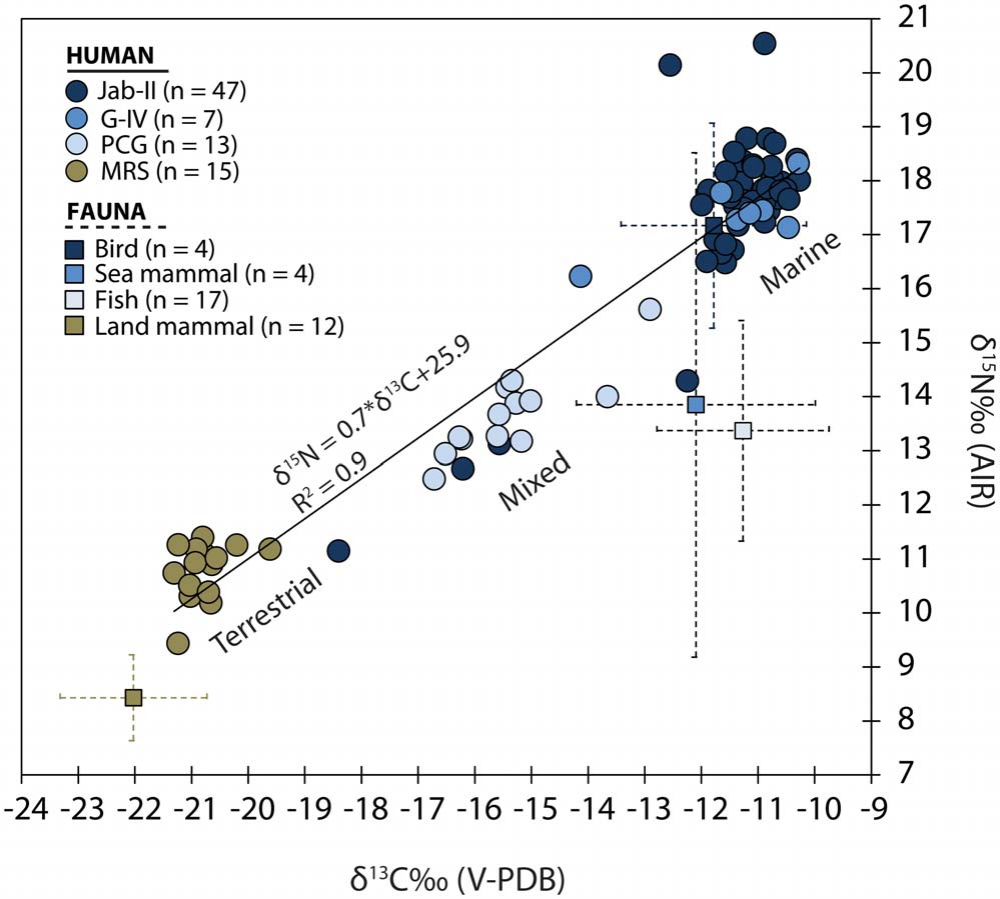
Bulk collagen δ^13^C and δ^15^N values. Distribution of human and faunal values from Jabuticabeira II (Jab-II), Galheta IV (G-IV), Piaçaguera (PCG) and Moraes (MRS). Fish values also include modern specimens.

Human δ^13^C and δ^15^N values show strong positive linear correlations (r = 0.95; R^2^ = 0.92; p<0.001; Tab. S2) and fall between the end-points derived from correcting the observed marine and C3 terrestrial fauna for isotopic fractionation (Fig. 3). Therefore the human isotope values can be largely explained by direct routing of both carbon and nitrogen from dietary protein to collagen, which implies that the diets contained sufficiently high protein [71]. The δ^13^C and δ^15^N values differ significantly among sites (p<0.001; Tab. 2). Lower δ^13^C and δ^15^N values were observed in inland individuals from MRS, as opposed to higher values of coastal pre-ceramic and ceramic individuals from Jab-II and G-IV, in agreement with preliminary isotopic studies [11], [21]. Individuals from PCG, instead, exhibit δ^13^C and δ^15^N values consistent with a mixed marine/C3 terrestrial diet.

**Table 2:** Average bone collagen δ^13^C and δ^15^N values of humans, including isotopic variability (Δδ%) and the number of individual analysed.

Site	δ^13^C%	Δδ^13^C%	δ^15^N%	Δδ^15^N%	N
MRS	–20.8±0.4	1.7	+10.8±0.5	2.0	15
PCG	–15.4±1.0	3.8	+13.7±0.8	3.1	13
Jab-II	–11.5±1.5	8.1	+17.4±1.6	9.4	47
G-IV	–11.4±1.2	3.8	+17.4±0.6	2.1	7

**Figure 4:**
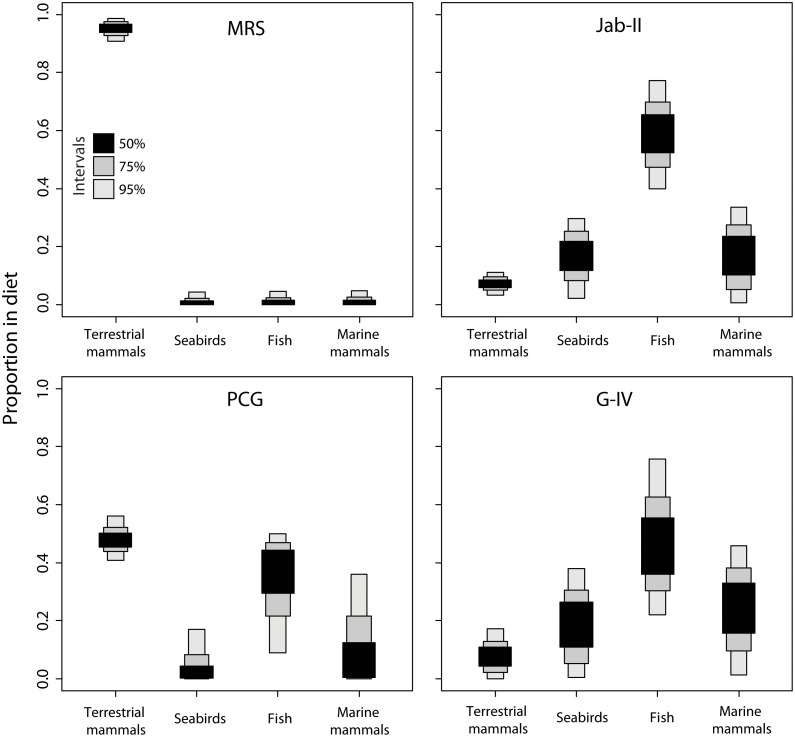
Bayesian-derived proportion of protein sources for archaeological site according to 95%, 75% and 50% of the dataset. Overlap of land mammal and fish in PCG show that human diet was based on marine as well as terrestrial items. This is not observed at MRS, Jab-II and G-IV.

**Figure 5:**
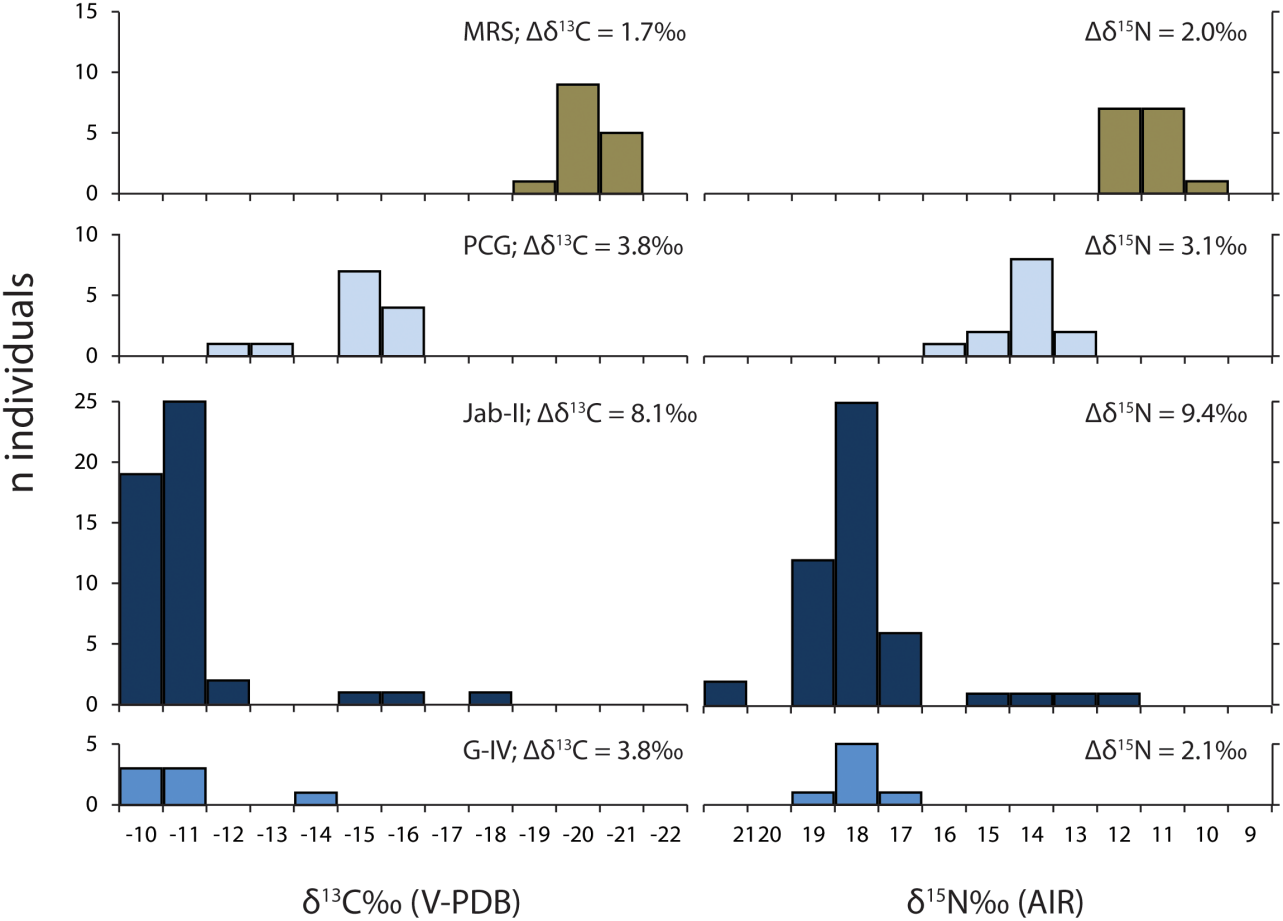
Bulk collagen δ^13^C and δ^15^N variability (Δδ) in inland and coastal populations. Note the large isotopic variability in humans from Jab-II.

The proportional contribution of different marine and C3 terrestrial animal resources to each individual’s diet can be crudely estimated through linear interpolation between the marine and terrestrial end-members. We also used a Bayesian model which generates possible dietary solutions from multiple dietary source categories. This model predicts that inland peoples acquired >90% of their protein from C3 terrestrial resources, whereas people on the coast were assimilating protein mainly from fish, up to 80%, along with some contribution from seabirds and sea mammals (Fig. 4). A large isotopic variability, however, was detected in coastal groups, in particular at Jab-II (Fig. 5) but it seems not to be related to sex and age (Tab. S3).

It is important to note that these estimations refer to the protein contribution to total dietary protein (by dry weight) and not the contribution to total diet. In this case, the bulk isotopic analyses are highly insensitive to the other dietary components, such as carbohydrate and lipid, which must have been consumed to some extent in order to avoid protein poisoning [72].

### Carbon Stable Isotope Analysis of Single Amino Acids

Stable carbon isotope values were obtained from 15 amino acids corresponding to 97.5% of the carbon atoms in collagen (Tab. S4). Mass balance calculations were used to estimate the δ^13^C of whole collagen from the measured individual amino acid δ^13^C values. These estimated values were strongly correlated with the observed bulk δ^13^C values (R^2^ = 0.98) and the offset between the estimated and observed measurements was <1% in all cases. The δ^13^C of individual amino acids were strongly and positively correlated with both the δ^13^C (R^2^ = >0.8) and δ^15^N (R^2^ = >0.7) values of whole collagen for both marine and C3 terrestrial consumers, confirming that both dispensable and non-dispensable amino acids were largely derived from a dietary protein source.

**Figure 6:**
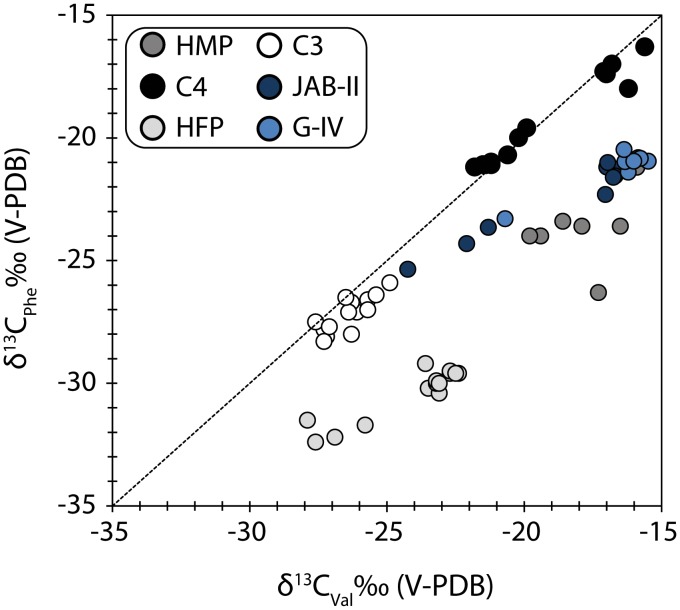
A biplot of phenylalanine and valine δ^13^C values. The biplot distinguishes two dietary groups at Jab-II and G-IV; those with mixed marine/C3 terrestrial animal diets and those consuming high marine protein (HMP). Data from Honch et al. [28] are reported for comparison and include C4, C3 and high marine (HMP) and freshwater protein (HFP) consumers.

Honch et al. [28] devised a method of interpreting dietary intake of individuals using a plot of δ^13^C values of phenylalanine (Phe) and valine (Val) of bone collagen hydrolysates. Following this method, the δ^13^C values again confirm the two dietary groups; those with mixed marine/C3 terrestrial resource diets (Jab-II, burials 17C, 24A, 102 and G-IV, burial 7) and all other samples as high marine protein (HMP) consumers (Fig. 6).

**Figure 7:**
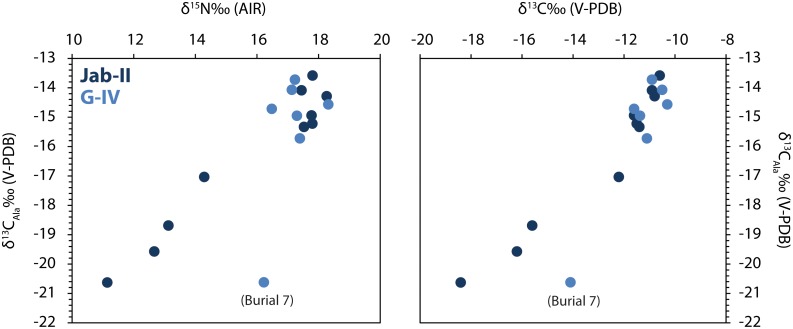
Bulk collagen δ^13^C and δ^15^N against alanine δ^13^C values for individuals from Jab-II and G-IV. Notice the strong positive correlation of alanine δ^13^C values with bulk collagen δ^13^C and δ^15^N. Exceptionally alanine is depleted in ^13^C in one individual from G-IV (burial 7) likely implying a larger contribution of C3 plant carbohydrate in the diet.

There are some interesting observations concerning the amino acid δ^13^C values of burial 7 at G-IV. In general the values for both dispensable and non-dispensable amino acids (Tab. S4) for this individual are intermediate between the mixed marine/C3 terrestrial consumers and the HMP consumers, indicating a mixed diet. Unexpectedly however, this individual has a lower δ^13^C value for alanine (Ala) compared to the others (Fig. 7). In the context of the other amino acid values, the low alanine value is difficult to explain. If the alanine were derived from a protein source with low δ^13^C values (e.g. C3 terrestrial or freshwater fish) we would expect other amino acids to be ^13^C depleted (especially Val and Phe; Fig. 6), which is not the case. Ala is a dispensable amino acid and can be directly routed from diet but is also readily synthesized from precursors originating in the first steps of glycolysis [73]. The latter are predominantly derived from dietary carbohydrates. Recently Choy et al. [73] noted that alanine values (in red blood cells and hair keratin of modern individuals) were strongly related to carbohydrate intake and not other dietary sources (i.e. meat, fish, marine mammals and corn products) whilst the other dispensable amino acid δ^13^C values (Pro, Glx, Asx, Ser and Gly) were not related to carbohydrate intake. We can interpret the δ^13^C alanine data from the individual in burial 7 at G-IV as an individual who had a long term diet that contained a higher amount of ^13^C depleted carbohydrate, e.g. from C3 plants, compared to the others. Apart from this exception, the carbon isotope analysis of individual amino acids confirms the bulk collagen analysis and suggests that the majority of individuals consumed adequate protein to supply the nearly all the carbon in collagen. Whether alanine δ^13^C values can be used as a carbohydrate marker in palaeodietary contexts warrants further testing.

### Organic Residue Analysis from Pottery at G-IV

**Figure 8:**
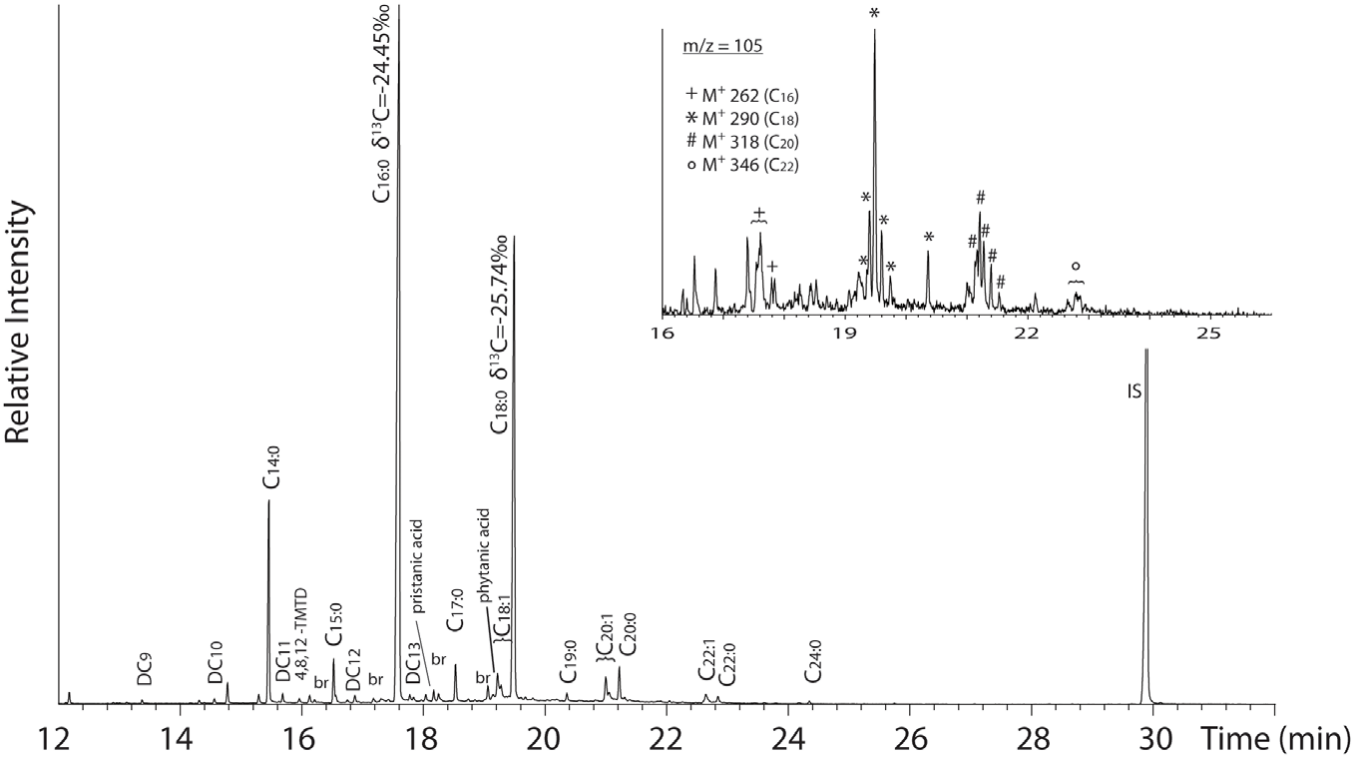
Partial total ion current chromatograms showing the methylated lipids extracted from a ceramic sherd (G18E). C*_n_*_:*x*_ - fatty acids with carbon length *n* and number of unsaturations *x*, DC_n_ - α,ω-dicarboxylic acids with carbon length *n,* br - branched chain acids, phytanic acid, pristanic acid, 4,8,12-TMTD - 4,8,12-tryimethyltridecanoic acid), IS - internal standard (*n*-hexatriacontane). *m/z* 105 ion chromatogram showing the presence of ω(ο-alkylphenyl)alkanoic acids with 16 (+), 18(*), 20(#) and 22 (ο) carbon atoms.

Molecular and isotopic compositions of adsorbed organic residues indicate that pottery vessels were used for the processing of marine products, along with plants and other animal resources. Overall the lipid preservation was poor, and the majority of samples contained only low levels (<0.5 μg mg^−1^) of palmitic and stearic acids. Nevertheless one sample (G18E) has a lipid profile consisting of medium- and long-chain saturated (C_14_–C_24_) and monounsaturated (C_18∶1_–C_22∶1_) fatty acids, isoprenoid fatty acids (4,8,12-trimethyltridecanoic acid and phytanic acid) and long-chain (C_18_–C_22_) ω-(o-alkylphenyl) fatty acids (Fig. 8, Tab. S5). Such a profile is characteristic of degraded aquatic oils, as established on contemporaneous pottery from this region [22]. A similar lipid distribution was found in G16P but with lesser preservation of ω-(o-alkylphenyl) fatty acids, preventing a clear confirmation of aquatic oils. This sample and G24P contain a series of triterpenes (m/z 189, 218) revealing the presence of plant resins which were also found in Taquara/Itararé pottery assemblages and were interpreted as a waterproofing coating [23]. Finally, one sample (G22P) had low quantities of very long chain fatty acids (up to C_28∶0_), traces of long chain dicarboxylic fatty acids (C_22_–C_24_) and isomers of the C_18_ ω-(o-alkylphenyl) fatty acid only. The latter compound is formed by heat alteration of polyunsaturated C_18_ fatty acids, which is consistent with a plant contribution in the profile. It also contains an unusually high concentration of C_12∶0_ which in this context could be derived from palm kernel oil [74], [75], although the difference in the fatty acid distribution as the various biomarkers present suggest a complex mixture of lipids from different origins.

The δ^13^C values (both C_16∶0_ and C_18∶0_≥–25%) of medium chain-length n-hexadecanoic (C_16∶0_) and *n*-octadecanoic (C_18∶0_) acids from 7 out of 11 pottery samples are within the range of marine oils reported in previous studies (Tab. S5). Lower δ^13^C values, in contrast, match those observed in modern pottery vessels used to process freshwater and non-ruminant animal fats and oils [30], [31]. There was no evidence for the processing of ruminants in pottery [59] despite the presence of cervids (*Mazama* sp., *Ozotoceros bezoarticus*) in the faunal assemblages [41]. Biomarkers associated with maize processing, e.g. *n*-dotriacontanol, were absent in all the vessels studied although there are doubts whether these would accumulate or preserve in sufficient quantities to allow identification [76].

Charred surface residues show δ^13^C and δ^15^N values ranging from −22.6% to −25.8% and from +6.7% to +12.7% respectively (Tab. S6). Samples enriched in ^13^C and depleted in ^15^N may tentatively indicate some contribution of C4 plants [77]. However the correlation between C4 plants (e.g. maize) and δ^13^C and δ^15^N values of charred deposit are not straightforward [78], [79], and our δ^13^C and δ^15^N results are also consistent with those observed in coastal areas of Northern Europe resulting from the processing of aquatic resources [30]. In spite of the complexity underlying food crust isotopic signatures, compound specific isotopic data from the same potsherds reinforces the interpretation that marine foods principally contributed to the isotopic signal of these charred deposits.

## Discussion

Marine and C3 terrestrial animals were the main sources of protein for coastal and inland sambaqui builders of S. Brazil between ∼6,700 and ∼1,700 cal BP. The isotopic gradient from the inland to the coast suggests the existence of confined catchment areas and/or selective targeting of specific resources [80]–[82], which is a common feature amongst sambaqui builders [11], [13], [20], [21] and other coastal populations in South America [81]. There is no isotopic evidence for the contribution of freshwater resources, which is consistent with the very low frequency of these remains in both mainland and coastal sites [11], [21]. Interestingly, some individuals at Jab-II have an unusually high intake of C3 terrestrial proteins, denoting some degree of population variability on the coast. Although the lack of significant isotopic differences between sexes and age is consistent with pervasive food sharing among these populations, the intra-population δ^13^C and δ^15^N variability at Jab-II may point to the presence of non-local individuals, as observed in other preceramic coastal populations [18], perhaps assimilated into the group through post-marital residential practices [83]; however isotopic variability may also be associated to food restrictions among members of the community [84].

Our results also revealed that the peoples at G-IV relied substantially on marine resources, to the same extent as the pre-ceramic coastal adapted populations. The isotopic results of diets at coastal sites are broadly supported by the rich archaeozoological evidence dominated by marine resources [21], [41], [85]. Collagen from individuals at Jab-II and G-IV are amongst the most enriched in ^13^C and ^15^N in the eastern coast of South America between ∼8,000 and ∼1,000 cal BP [20], [21], [81], [86]–[97].

The carbon isotope signature of individual amino acids indicate a minor contribution of plants to the diet of some individuals, including those that post-date the adoption of pottery on the coast. The occurrence of mortars and plant macro-remains at several, but not all, sites [1], [4], [5], [98], along with variable degree of caries, starch grains in dental calculus [5], [6] and dental wear [99] also indicate that plants made a contribution to the diets of some coastal groups at this time [1], [3], [100]. Furthermore preliminary studies have also successfully extracted phytoliths and starch grains from charred deposits of pottery from G-IV, and one individual provided amino acid (alanine) δ^13^C values suggestive of a larger intake of C3 carbohydrates. However, the bulk collagen isotope analysis indicates that plants were unlikely to be major dietary staples for these coastal groups, rather the diet was protein-rich and oriented toward marine resources.

The continuity in coastal exploitation is further supported by molecular and isotopic results from organic residues preserved in pottery. These data attest to the use of pottery for the processing of animal products, including marine organisms. Hansen and Schmitz [23] achieved similar results from coeval sites in southern Brazil, revealing that pottery was commonly used for the manipulation of marine resources. Combined results therefore indicate that the adoption of pottery in coastal areas is not directly connected with the imposition of food production and did not affect the proportional contribution of marine resources to the diet of coastal populations. Small ceramic vessels had most likely a ritual and symbolic utility [16], [41]. Therefore, the molecular and isotopic analyses provide new direct evidence of the importance of marine resources in symbolic spheres. This is an interesting finding as it may have been assumed that novel or exotic cultivated plants would have had a more ritual and symbolic role in cuisine and therefore would have been more visible in the pottery contents.

## Conclusion

Maritime adaptations sustained South American pre-Columbian populations since the late Pleistocene [101] and stable isotope studies reveal the crucial role of aquatic resources to several Holocene coastal groups (e.g. [86]–[92]), even during the intensification of food production (e.g. [82], [102]–[108]). However these direct lines of evidence are strongly biased towards archaeological records along the western and south-eastern coast of South America. Here we have extended the information to the subtropical Atlantic rainforest coast of Brazil. The isotope results show that it is highly unlikely that these coastal populations relied on plant carbohydrate as a major dietary source. Instead, we demonstrate the strong dependence of marine animal resources, despite the decline of monumental shell mound building and the arrival of a new subsistence strategy at ∼1,500 cal BP, involving domesticated plants and pottery technology, from inland areas. Therefore our results imply that the productive maritime economy was highly resilient to social and cultural change. It remains to be assessed if ceramic producing populations on the coast were directly descended from indigenous coastal foragers or immigrants from the highlands who, having reached the coast, oriented their economy toward aquatic resources. However the resilient character of this subsistence system is further expressed by its flexibility. Rather than transforming the coastal economy, as observed in the Atlantic coasts of Europe [109], [110], the adoption of pottery was incorporated into marine focused subsistence strategies [30]. These results emphasize how the archaeological record offers a unique and exceptional opportunity to illuminate the longstanding trajectory of New World maritime adaptations, which still today play a pivotal role to coastal populations in Latin America [111].

## Supporting Information

Table S1 **Carbonate ν_1_ (C) to phosphate ν_1_ (P) intensity ratios, full width at half maximum (FWHM) of the phosphate ν_1_ (P) band and organic (C-H stretch) to phosphate ν_1_ (P) intensity ratios determined for each averaged spectrum as a function of the sample type.** Samples are ordered from youngest to oldest with data acquired using the same Raman confocal settings across all samples. Modern lamb bone is justified as an appropriate control due to the similarities in sheep and human bone as per the RS study of Rehman et al. [52].
(DOCX)

Table S2 **Bone collagen δ^13^C and δ^15^N values of humans. Also show the age class or the relative age (young, adult) and the sex (F: female, M: male).**
(DOCX)

Table S3 **One-way ANOVA showing a general lack of significant isotopic differences between sexes and age at MRS, Jab-II and PCG**. Data from G-IV was not sufficient to be tested statistically. Because of the limited information about the age, individuals from MRS and PCG were sorted out in two categories: <36 and >36 years old. At Jab-II, individuals belonging to the age class 11–20 years old show higher δ^15^N values (mean 19.1±1.0%, n = 3) than individuals belonging to the age class 36–50 yrs (mean 17.3±0.9%, n = 13).
(DOCX)

Table S4 **Collagen amino acid δ^13^C values for humans from Jab-II (n = 10) and G-IV (n = 7).**
(DOCX)

Table S5 **Ceramic sherds selected for lipid analysis by GCMS and GC-c-IRMS.** FA (Cx:y) - fatty acids with carbon length x and number of unsaturations y, br -branched chain acids, phy- phytanic acid, TMTD - 4,8,12-trimethyltridecanoic acid. APFA (Cn) - ω-(o-alkylphenyl) alkanoic acids with carbon length n. tr - trace. DCx - α,ω-dicarboxylic acids with carbon length x. P - interior, E - exterior. Aquatic oils are interpreted from the presence of isomers of APFA (C_20_ or C_22_) and at least one isoprenoid fatty acids (pri, phy or TMTD). Resins are interpreted from the presence of triterpenes. Plant oils are interpreted from the presence of long chain fatty acids, dicarboxylique acids and the presence of isomers of C_18_ APFA. A high abundance of C_12∶0_ could be consistent with Palm Kernel. Aquatic (marine) fats are defined on the isotopic characteristics of the C_16_ and C_18_ saturated fatty acids.
(DOCX)

Table S6 **Bulk isotope characteristics of charred deposits from the interior of potsherds from G-IV.**
(DOCX)

## References

[1] DeBlasis PAD, Fish SK, Gaspar MD, Fish PR (1998) Some references for the discussion of complexity among the Sambaqui moundbuilders from the southern shores of Brasil. Rev Arqueol Amer 15: 75–105.

[2] Figuti L (1993) O homem pré-histórico, o molusco e o sambaqui. Revista do Museu de Arqueologia e Etnologia USP 3: 67–80.

[3] Neves WA, Wesolowski V (2002) Economy, nutrition and disease in prehistoric coastal Brazil: a case study from the state of Santa Catarina. In: Steckel RH, Rose JC editors. The Backbone of History: Health and Nutrition in the Western Hemisphere. Cambridge University Press, 376–405.

[4] Scheel-Ybert R, Eggers S, Wesolowski V, Petronilho CC, Boyadjian CH, et al (2003) Novas perspectivas na reconstituição do modo de vida dos sambaquieiros: uma abordagem multidisciplinar. Revista Arqueologia 16: 109–137.

[5] Scheel-Ybert R, Eggers S, Wesolowski V, Petronilho CC, Boyadjian CH, et al (2009) Subsistence and lifeway of coastal Brazilian moundbuilders. In: Treballs d’Etnoarqueologia Capparelli A, Chevalier A, Piqué R, editors. La alimentación en la América precolombina y colonial: una aproximación interdisciplinaria. 7: 37–53.

[6] Boyadjian CHC, Eggers S, Reinhard K (2007) Dental wash: a problematic method for extracting microfossils from teeth. Journal of Archaeological Science 34: 1622–1628.

[7] Wesolowski V, Mendonça de Souza SMF, Reinhard KJ, Ceccantini G (2010) Evaluating microfossil content of dental calculus from Brazilian sambaquis. Journal of Archaeological Science 37: 1326–1338.

[8] Okumura MMM, Eggers S (2012) Living and eating in coastal Brazil during prehistory. In: Collard D, Morris J, Perego E, editors. Food and Drink in Archaeology 3: Prospect Books. 55–64.

[9] Figuti L, Plens CR, DeBlasis PAD (2013) Small sambaquis and big chronologies: shellmound building and hunter-gatherers in Neotropical highlands. Radiocarbon 55(2–3): 1215–1221.

[10] Plens CR (2009) O papel dos amontoados de conchas no sambaqui fluvial. Revista de Arqueologia 22(2): 77–93.

[11] Plens CR (2010) Animals for humans in life and death. Museu de Arqueologia e Etnologia 20: 31–52.

[12] Eggers S, Parks M, Grupe G, Reinhard KJ (2011) Paleoamerican diet, migration and morphology in Brazil: archaeological complexity of the earliest Americans. PLoS ONE 6(9): e23962 10.1371/journal.pone.0023962 21935369 PMC3173364

[13] DeBlasis PAD, Kneip A, Scheel-Ybert R, Giannini P, Gaspar M (2007) Sambaquis e paisagem dinâmica natural e arqueologia regional do sul do Brasil. Arqueologia Suramericana/Arqueologia Sul-Americana 3 1: 29–61.

[14] Iriarte J, Behling H (2007) The expansion of Araucaria forest in the southern Brazilian highlands during the last 4000 years and its implications for the development of the Taquara/Itararé Tradition. Environmental archaeology 12(2): 115–127.

[15] Noelli F (2000) A ocupação humana na região sul do Brasil: arqueologia, debates e perspectivas 1972–2000. Revista da USP 44: 218–269.

[16] Iriarte J, Gillam JC, Marozzi O (2008) Monumental burials and memorial feasting: an example from the southern Brazilian highlands. Antiquity 82(318): 947–961.

[17] González EMR (1998) Regional pottery-making groups in southern Brazil. Antiquity 72(277): 616–624.

[18] Bastos MQR, Mendonça de Souza, SMF, Santos RV, Lima BAF, Santos RV, et al (2011) Human mobility on the Brazilian coast: an analysis of strontium isotopes in archaeological human remains from Forte Marechal Luz sambaqui. Anais da Academia Brasileira de Ciências 83(2): 731–743. 21670891 10.1590/s0001-37652011000200030

[19] Schmitz PI, Rosa AO, Izidro JM, Haubert F, Krever MLB, et al (1999) Içara: Um jazigo mortuário no litoral de Santa Catarina. Pesquisas, Antropologia 55: 01–164.

[20] De Masi MAN (2001) Pescadores coletores da costa sul do Brasil. Pesquisas 57: 1–136.

[21] Villagran XS, Klokler D, Peixoto S, DeBlasis PAD, Giannini PCF (2011) Building coastal landscapes: zooarchaeology and geoarchaeology of Brazilian shell mounds. Journal of Island & Coastal Archaeology 6: 211–234.

[22] Hansel FA, Copley MS, Madureira LAS, Evershed RP (2004) Thermally produced omega-(o-alkylphenyl)alkanoic acids provide evidence for the processing of marine products in archaeological pottery vessels. Tetrahedron Letters 45: 2999–3002.

[23] Hansel FA, Schmitz PI (2006) Classificação e interpretação dos resíduos orgânicos preservados em fragmentos de cerâmica arqueológica por cromatografia gasosa e cromatografia gasosa-espectrometria de massas. Pesquisas 63: 81–112.

[24] Schwarcz HP, Schoeninger MJ (1991) Stable isotope analyses in human nutritional ecology. Yearbook of Physical Anthropology 34: 283–321.

[25] Schulting RJ (2011) Mesolithic-Neolithic transitions: an isotopic tour through Europe. In: Pinhasi R, Stock J editors. The bioarchaeology of the transition to agriculture. New York: Wiley-Liss. 17–41.

[26] Fogel M, Tuross N (2003) Extending the limits of paleodietary studies of humans with compound specific carbon isotope analysis of amino acids. Journal of Archaeological Science 30(5): 535–545.

[27] Choy K, Smith CI, Fuller BT, Richards MP (2010) Investigation of amino acid δ^13^C signatures in bone collagen to reconstruct human palaeodiets using liquid chromatography–isotope ratio mass spectrometry. Geochimica et Cosmochimica Acta 74: 6093–6111.

[28] Honch NV, McCullagh JSO, Hedges REM (2012) Variation of bone collagen amino acid δ^13^C values in archaeological humans and fauna with different dietary regimes: developing frameworks of dietary discrimination. American Journal of Physical Anthropology 148: 495–511. 22610935 10.1002/ajpa.22065

[29] Evershed RP, Bull ID, Corr LT, Crossman ZM, Vandongen BE, et al. (2007) Compound-specific stable isotope analysis in ecology and paleoecology. In: Michener R, Lajtha K, editors. Stable isotopes in ecology and environmental science. Blackwell Publishing Ltd, Singapore, 480–540.

[30] Craig OE, Steele VJ, Fischer A, Hartz S, Andersen SH, et al (2011) Ancient lipids reveal continuity in culinary practices across the transition to agriculture in Northern Europe. Proc. Natl Acad. Sci. USA 108: 17910–17915. 10.1073/pnas.1107202108 PMC3207664 22025697

[31] Craig OE, Saul H, Lucquin A, Nishida Y, Taché K, et al (2013) Earliest evidence for the use of pottery. Nature 496: 351–354. 23575637 10.1038/nature12109

[32] De Masi MAN (2009) Aplicações de isótopos estáveis de ^18^/^16^O, ^13^/^12^C e ^15^/^14^N em estudos de sazonalidade, mobilidade e dieta de populações pré-históricas no sul do Brasil. Revista de Arqueologia 22(2): 55–76.

[33] Figuti L, Mendonça CA, Porsani JL, Rocha EB, DeBlasis PAD, et al. (2004) Relatório final FAPESP: Investigação arqueológica e geofísica nos sambaquis fluviais no vale Ribeira de Iguape estado de São Paulo –FAPESP n° 1999/12684–2.

[34] Eggers S, Petronilho CC, Brandt K, Jericó-Daminello C, Filippini J, Reinhard KJ (2008) How does a riverine setting affect the lifestyle of shellmound builders in Brazil? HOMO–Journal of Comparative Human Biology 59: 405–427. 10.1016/j.jchb.2008.04.005 19027113

[35] Fischer PF (2012) Os moleques do morro e os moleques da praia: estresse e mortalidade em um sambaqui fluvial (Moraes, vale do Ribeira de Iguape, SP) e em um sambaqui litorâneo (Piaçaguera, Baixada Santista, SP). Unpublished thesis. Universidade de São Paulo.

[36] Garcia C (1970) Meios de subsistência de populações pré-históricas no litoral do estado de São Paulo. Unpublished thesis. Universidade de São Paulo.

[37] Garcia C (1972) Estudo comparado das fontes de alimentacao de duas populacoes pre-historicas do litoral paulista. Unpublished PhD thesis. Universidade de São Paulo.

[38] Uchôa DP (1970) O sítio arqueológico de Piaçagüera: aspectos gerais. Unpublished thesis. Universidade de São Paulo.

[39] Okumura MMM, Eggers S (2005) The people of Jabuticabeira II: reconstruction of the way of life in a Brazilian shellmound. HOMO - Journal of Comparative Human Biology 55: 263–281. 15803771 10.1016/j.jchb.2004.10.001

[40] Klokler D (2008) Food for body and soul: mortuary ritual in shell mounds (Laguna - Brazil). Unpublished PhD thesis. The University of Arizona.

[41] DeBlasis PAD, Farias DS, Kneip A (in press) Velhas tradições e gente nova no pedaço: perspectivas longevas de arquitetura funerária na paisagem do litoral sul catarinense. Revista do Museo de Arqueologia e Etnologia da USP.

[42] Friedli H, Lötscher H, Oeschger H, Siegenthaler U, Stauffer B (1986) Ice core record of the ^13^C/^12^C ratio of atmospheric CO_2_ in the past two centuries. Nature 234(20): 237–238.

[43] Ambrose SH, Norr L (1993) Experimental evidence for the relationship of the carbon isotope ratios of whole diet and dietary protein to those of bone collagen and carbonate. In: Lambert JB, Grupe G, editors. Prehistoric Human Bone: Archaeology at the Molecular Level. Springer-Verlag, Berlin, 1–37.

[44] Jim S, Ambrose SH, Evershed RP (2004) Stable carbon isotopic evidence for differences in the dietary origin of bone cholesterol, collagen and apatite: implications for their use in palaeodietary reconstruction. Geochimica et Cosmochimica Acta 68(1): 61–72.

[45] Jim S, Jones V, Ambrose SH, Evershed RP (2006) Quantifying dietary macronutrient sources of carbon for bone collagen biosynthesis using natural abundance stable carbon isotope analysis. British Journal of Nutrition 95: 1055–1062. 16768826 10.1079/bjn20051685

[46] Schwarcz HP (2000) Some biochemical aspects of carbon isotopic paleodiet studies. In: Ambrose SH, Katzenberg MA, editors. Biogeochemical approaches to paleodietary analysis. New York: Kluwer Academic/Plenum, 189–209.

[47] Schoeninger MJ (2009) Stable isotope evidence for the adoption of maize agriculture. Current Anthropology 50(5): 633–640. 20642150 10.1086/605111

[48] Fernandes R, Nadeau MJ, Grootes PM (2012) Macronutrient-based model for dietary carbon routing in bone collagen and bioapatite. Archaeol Anthropol Sci 4: 291–301.

[49] Hedges REM (2002) Bone diagenesis: an overview of processes. Archaeometry 44(3): 319–328.

[50] Wright LE, Schwarcz HP (1996) Infrared and isotopic evidence for diagenesis of bone apatite at Dos Pilas, Guatemala: palaeodietary implications. Journal of Archaeological Science 23: 933–944.

[51] van Klinken GJ (1999) Bone collagen quality indicators for palaeodietary and radiocarbon measurements. Journal of Archaeological Science 26: 687–695.

[52] Rehman I, Smith R, Hench LL, Bonfield W (1995) Structural evaluation of human and sheer, bone and comparison with synthetic hydroxyapatite by FT-Raman spectroscopy. Journal of Biomedical Materials Research 29: 1287–1294. 8557731 10.1002/jbm.820291016

[53] Awonusi A, Morris MD, Tecklenburg MMJ (2007) Carbonate assignment and calibration in the Raman Spectrum of Apatite. Calcified Tissue International 81: 46–52. 17551767 10.1007/s00223-007-9034-0

[54] Craig OE, Biazzo M, Colonese AC, Di Giuseppe Z, Martinez-Labarga C, et al (2010) Stable isotope analysis of Late Upper Palaeolithic human and faunal remains from Grotta del Romito (Cosenza), Italy. Journal of Archaeological Science 37: 2504–2512.

[55] Smith CI, Fuller B T, Choy K, Richards MP (2009) A three-phase liquid chromatographic method for δ^13^C analysis of amino acids from biological protein hydrolysates using liquid chromatography-isotope ratio mass spectrometry. Anal Biochem 390: 165–172. 19379706 10.1016/j.ab.2009.04.014

[56] Evershed RP, Heron C, Goad LJ (1990) Analysis of organic residues of archaeological origin by high-temperature gas chromatography and gas chromatography - mass spectrometry. Analyst 115: 1339–1342.

[57] Craig OE, Forster M, Andersen AH, Koch E, Crombé P, et al (2007) Molecular and isotopic demonstration of the processing of aquatic products in Northern European prehistoric pottery. Archaeometry 49(1): 135–152.

[58] Reber EA, Dudd SN, van der Merwe NJ, Evershed RP (2003) Direct detection of maize in pottery residues via compound specific stable carbon isotope analysis. Antiquity 78(301): 682–691.

[59] Craig OE, Allen RB, Thompson A, Stevens RE, Steele VJ, Heron C (2012) Distinguishing wild ruminant lipids by gas chromatography/combustion/isotope ratio mass spectrometry. Rapid Communications in Mass Spectrometry 26: 2359–2364. 22956328 10.1002/rcm.6349

[60] Parnell AC, Inger R, Bearhop S, Jackson AL (2010) Source partitioning using stable isotopes: coping with too much variation. PLoS ONE 5(3): 9672–9672. 10.1371/journal.pone.0009672 PMC2837382 20300637

[61] Richards MP, Hedges REM (1999) Stable isotope evidence for similarities in the types of marine foods used by late Mesolithic humans at sites along the Atlantic coast of Europe. Journal of Archaeological Science 26: 717–722.

[62] Hammer Ø, Harper DAT, Ryan PD (2001) PAST: paleontological statistics software package for education and data analysis. Palaeontologia Electronica 4(1): 1–9.

[63] McCormac FG, Hogg AG, Blackwell PG, Buck CE, Higham TFG, Reimer PJ (2004) SHCal04 southern Hemisphere calibration, 0–11.0 cal kyr BP. Radiocarbon 46(3): 1087–1092.

[64] Bronk Ramsey C (2009) Bayesian analysis of radiocarbon dates. Radiocarbon 51(1): 337–360.

[65] Thomas DB, Fordyce RE, Frew RD, Gordon KC (2007) A rapid, non-destructive method of detecting diagenetic alteration in fossil bone using Raman spectroscopy. Journal of Raman Spectroscopy 38: 1533–1537.

[66] King CL, Tayles N, Gordon KC (2011) Re-examining the chemical evaluation of diagenesis in human bone apatite. Journal of Archaeological Science 38: 2222–2230.

[67] Nielsen-Marsh C, Gernaey A, Turner-Walker G, Hedges R, Pike A, Collins C (2000) Chemical degradation of bone. In: Cox M, Mays S editors. Human osteology in archaeology and forensic sciences. London: Greenwich Medical Media, 439–453.

[68] Morris MD, Mandair GS (2011) Raman assessment of bone quality. Clinical Orthopaedics and Related Research 469: 2160–2169. 21116756 10.1007/s11999-010-1692-y PMC3126952

[69] Edwards HGM, Farwell DW, de Faria DLA, Monteiro AMF, Afonso MC, et al (2001) Raman spectroscopy study of 3000-year-old human skeletal remains from a sambaqui, Santa Catarina, Brazil. Journal of Raman Spectroscopy 32: 17–22.

[70] Pestle WJ, Colvard M (2012) Bone collagen preservation in the tropics: a case study from ancient Puerto Rico. Journal of Archaeological Science 39(7): 2079–2090.

[71] Craig OE, Bondioli L, Fattore L, Higham T, Hedges R (2013a) Evaluating marine diets through radiocarbon dating and stable isotope analysis of victims of the AD 79 eruption of Vesuvius. American Journal of Physical Anthropology DOI:10.1002/ajpa.22352. 24000142

[72] Noli D, Avery G (1988) Protein poisoning and coastal subsistence. Journal of Archaeological Science 15: 395–401.

[73] Choy K, Nash SH, Kristal AR, Hopkins S, Boyer BB, O’Brien DM (2013) The carbon isotope ratio of alanine in red blood cells is a new candidate biomarker of sugar-sweetened beverage intake. Journal of Nutrition 143: 878–884. 23616504 10.3945/jn.112.172999 PMC3652884

[74] Coimbra MC, Jorge N (2011) Characterization of the Pulp and Kernel Oils from *Syagrus oleracea*, *Syagrus romanzoffiana*, and *Acrocomia aculeate* . Journal of Food Science 76: C1156–C1161. 22417579 10.1111/j.1750-3841.2011.02358.x

[75] Coimbra MC, Jorge N (2012) Fatty acids and bioactive compounds of the pulps and kernels of Brazilian palm species, guariroba (*Syagrus oleraces*), jerivá (*Syagrus romanzoffiana*) and macaúba (*Acrocomia aculeata*). Journal of the Science of Food and Agriculture 92: 679–684. 21922463 10.1002/jsfa.4630

[76] Reber EA, Evershed RP (2004) How did Mississippians prepare maize? The application of compound-specific carbon isotope analysis to absorbed pottery residues from several Mississippi Valley sites. Archaeometry 46(1): 19–33.

[77] Morton JD, Schwarcz HP (2004) Palaeodietary implications from stable isotopic analysis of residues on prehistoric Ontario ceramics. Journal of Archaeological Science 31: 503–517.

[78] Hart JP, Lovis WA, Schulenberg JK, Urquhart GR (2007) Paleodietary implications from stable carbon isotope analysis of experimental cooking residues. Journal of Archaeological Science 34: 804–813.

[79] Hart JP, Lovis WA, Jeske RJ, Richards JD (2012) The potential of bulk δ^13^C on encrusted cooking residues as independent evidence for regional maize histories. American Antiquity 77(2): 315–325.

[80] Walker PL, DeNiro MJ (1986) Stable nitrogen and carbon isotope ratios in bone collagen as indices of prehistoric dietary dependence on marine and terrestrial resources in southern California. American Journal of Physical Anthropology 715–761. 10.1002/ajpa.1330710107 3777147

[81] Borrero LA, Guichón R, Tykot R, Kelly J, Prieto A, Cárdenas P (2001) Dieta a partir de isótopos estables en restos óseos humanos de Patagonia austral. Estado actual y perspectivas. Anales Instituto de la Patagonia 29: 119–127.

[82] Tomczak PD (2003) Prehistoric diet and socioeconomic relationships within the Osmore Valley of southern Peru. Journal of Anthropological Archaeology 22: 262–278.

[83] Hubbe M, Neves WA, Castro de Oliveira E, Strauss A (2009) Postmarital residence practice in southern Brazilian coastal groups: Continuity and change. Latin American Antiquity 20(2): 267–278.

[84] Begossi A, Hanazaki N, Ramos RM (2004) Food chain and the reasons for fish taboos among amazonian and atlantic forest fishers (Brazil). Ecological Applications 14(5): 1334–1343.

[85] de Castilho PV (2008) Utilization of cetaceans in shell mounds from the southern coast of Brazil. Quaternary International 180: 107–114.

[86] Gómez Otero J, Belardi JB, Tykot R, Grammer S (2000) Dieta y poblaciones humanas en la costa norte del Chubut (Patagonia Argentina). In Desde el paìs de los Gigantes. Perspectivas arqueológicas en Patagonia. Universidad Nacional de la Patagonia Austral: Río Gallegos, 109–122.

[87] Guichón RA, Borrero LA, Prieto IA, Cardenas P, Tykot R (2001) Nuevas determinaciones de isótopos estables para Tierra del Fuego. Revista Argentina de Antropología Biológica 3(1): 113–126.

[88] Yesner DR, Torres MJF, Guichon RA, Borrero LA (2003) Stable isotope analysis of human bone and ethnohistoric subsistence patterns in Tierra del Fuego. Journal of Anthropological Archaeology 22: 279–291.

[89] Panarello H, Zangrando F, Tessone A, Kozameh LF, Testa N (2006) Comparative analysis of human diets between the Beagle Channel region and Peninsula Mitre: perspectives from stable isotopes. Magallania 34(2): 37–46.

[90] Favier Dubois C, Borella F, Tykot R (2009) Explorando tendencias en el uso humano del espacio y los recursos en el litoral rionegrino (Argentina) durante el Holoceno medio y tardío. In: Salemme M, Santiago F, Álvarez M, Piana E, Vázquez M, Mansur E, editors. Arqueología de la Patagonia - Una mirada desde el último confín. Editorial Utopías, Ushuaia, 985–998.

[91] Moreno E, Zangrando AF, Tessone A, Castro A, Panarello H (2011) Isótopos estables, fauna y tecnología en el estudio de los cazadores-recolectores de la costa norte de santa Cruz. Magallania 39(1): 265–276.

[92] Borrero LA, Barberena R, Franco NV, Charlin J, Tykot RH (2009) Isotopes and rocks: geographical organisation of southern Patagonian hunter-gatherers. International Journal of Osteoarchaeology 19: 309–327.

[93] Orquera LA, Piana EL (1996) El sitio Shamakush I. Relaciones de la Sociedad Argentina de Antropología. XXI: 215–265.

[94] Martínez G, Zangrando AF, Prates L (2009) Isotopic ecology and human palaeodiets in the lower basin of the Colorado River, Buenos Aires province, Argentina. International Journal of Osteoarchaeology 19: 281–296.

[95] Politis GG, Scabuzzo C, Tykot R (2009) An approach to Pre-Hispanic diets in the Pampas during the early/middle Holocene. International Journal of Osteoarchaeology 19: 266–280.

[96] Santiago F, Salemme M, Suby J, Guichón R (2011) Restos humanos en el norte de Tierra del Fuego. Aspectos contextuales, dietarios y paleopatológicos. Intersecciones en Antropología 12: 147–162.

[97] Borrero LA, Barberena R (2006) Hunter-gatherer home ranges and marine resources. An archaeological case from southern Patagonia. Current Anthropology 47(5): 855–867.

[98] Scheel-Ybert R, Dias OF (2007) Corondó: palaeoenvironmental reconstruction and palaeoethnobotanical considerations in a probable locus of early plant cultivation (south-eastern Brazil). Environmental Archaeology 12(2): 129–138.

[99] Turner II C, Machado LMC (1983) A new dental wear pattern and evidence for high carbohydrate consumption in a Brazilian archaic skeletal population. American Journal of Physical Anthropology 61: 125–130. 6869509 10.1002/ajpa.1330610113

[100] Boyadjian CHC (2012) Análise e identificação de microvestígios vegetais de cálculo dentário para a reconstrução de dieta sambaquieira: estudo de caso de Jabuticabeira II, SC. Unpublished thesis, Universidade de São Paulo.

[101] Sandweiss DH, McInnis H, Burger RL, Cano A, Ojeda B, et al (1998) Quebrada Jaguay: early South American maritime adaptations. Science 281: 1830–1832. 9743490 10.1126/science.281.5384.1830

[102] van der Merwe NJ, Lee-Thorp JA, Raymond JS (1993) Light, stable isotopes and the subsistence base of Formative cultures at Valdivia, Ecuador. In: Lambert JB, Grupe G, editors. Prehistoric Human Bone: Archaeology at the Molecular Level. Berlin: Springer-Verlag, 63–97.

[103] Tykot RH, Staller JE (2002) The importance of early maize agriculture in coastal Ecuador: new data from La Emerenciana. Current Anthropology 43(4): 666–677.

[104] Tykot RH, Burger R, van der Merwe NJ (2006) The importance of maize in initial period and early horizon Peru. In: Staller JE, Tykot RH, Benz BF editors. Histories of maize: multidisciplinary approaches to the prehistory, linguistics, biogeography, domestication, and evolution of maize. Academic Press, Burlington, Massachusetts, 187–197.

[105] Torres-Rouff C, Pestle WJ, Gallardo F (2012) Eating fish in the driest desert in the world: osteological and biogeochemical analyses of human skeletal remains from the San Salvador cemetery: North Chile. Latin American Antiquity 23(1): 51–69.

[106] Aufderheide AC (1994) Contribution of chemical dietary reconstruction to the assessment of adaptation by ancient highland immigrants (Alto Ramirez) to coastal conditions at Pisagua, North Chile. Journal of Archaeological Science 21: 515–524.

[107] White CD, Nelson AJ, Longstaffe FJ, Grupe G, Jung A (2009) Landscape bioarchaeology at Pacatnamu, Peru: inferring mobility from δ^13^C and δ^15^N values of hair. Journal of Archaeological Science 36: 1527–1537.

[108] Knudson KJ, Pestle WJ, Torres-Rouff C, Pimentel G (2010) Assessing the life history of an Andean traveller through biogeochemistry: stable and radiogenic isotope analyses of archaeological human remains from Northern Chile. International Journal of Osteoarchaeology 22(4): 435–451.

[109] Richards MP, Schulting RJ, Hedges REM (2003) Sharp shift in diet at onset of Neolithic. Nature 425: 366. 14508478 10.1038/425366a

[110] Cramp LJE, Jones J, Sheridan A, Smyth J, Whelton H, et al (2014) Immediate replacement of fishing with dairying by the earliest farmers of the northeast Atlantic archipelagos. Proc. R. Soc. B 2014 281: 20132372 10.1098/rspb.2013.2372. PMC4027381 24523264

[111] Begossi A (2010) Small-scale fisheries in Latin America: Management models and challenges. MAST 9: 5–12.

[112] Movasaghi Z, Rehman S, Rehman IU (2007) Raman spectroscopy of biological tissues, Applied Spectroscopy Reviews. 42: 493–541.

