# Correction: Long-Term Resilience of Late Holocene Coastal Subsistence System in Southeastern South America

**DOI:** 10.1371/journal.pone.0107034

**Published:** 2014-08-28

**Authors:** The PLOS ONE Staff

The isotopic values throughout the manuscript are incorrectly marked as %. All isotopic values are reported in ‰.
