# Supplementary material for: Long-Term Resilience of Late Holocene Coastal Subsistence System in Southeastern South America
Source: PLoS One. 2014 Apr 9;9(4):e93854. doi: 10.1371/journal.pone.0093854 (PMC3981759; doi:10.1371/journal.pone.0093854)
Supplement: Table S1 — Carbonate ν1 (C) to phosphate ν1 (P) intensity ratios, full width at half maximum (FWHM) of the phosphate ν1 (P) band and organic (C-H stretch) to phosphate ν1 (P) intensity ratios determined for each averaged spectrum as a function of the sample type. Samples are ordered from youngest to oldest with data acquired using the same Raman confocal settings across all samples. Modern lamb bone is justified as an appropriate control due to the similarities in sheep and human bone as per the RS study of Rehman et al. [52]. (DOCX) [file pone.0093854.s001.docx]

| **Samples** | **C/P**  **Intensity Ratio** | **FWHM**  **ν_1_ P band** | **CH/P**  **Intensity Ratio** |
| --- | --- | --- | --- |
| Modern | 0.190 ± 0.003 | 16.5 ± 0.2 | 0.83 ± 0.01 |
| G-IV | 0.52 ± 0.03 | 11.4 ± 0.5 | 0.67 ± 0.03 |
| JABII | 0.45 ± 0.02 | 12.9 ± 0.5 | 0.47 ± 0.02 |
| PCG | 0.71 ± 0.03 | 13.1 ± 0.8 | 0.64 ± 0.03 |
| MRS | 0.36 ± 0.01 | 13.8 ± 0.4 | 0.33 ± 0.01 |

**Table S1**: **Carbonate ν_1_ (C) to phosphate ν_1_ (P) intensity ratios, full width at half maximum (FWHM) of the phosphate ν_1_ (P) band and organic (C-H stretch) to phosphate ν_1_ (P) intensity ratios determined for each averaged spectrum as a function of the sample type**. Samples are ordered from youngest to oldest with data acquired using the same Raman confocal settings across all samples. Modern lamb bone is justified as an appropriate control due to the similarities in sheep and human bone as per the RS study of Rehman et al. [52].
