# Supplementary material for: Long-Term Resilience of Late Holocene Coastal Subsistence System in Southeastern South America
Source: PLoS One. 2014 Apr 9;9(4):e93854. doi: 10.1371/journal.pone.0093854 (PMC3981759; doi:10.1371/journal.pone.0093854)
Supplement: Table S2 — Bone collagen δ13C and δ15N values of humans. Also show the age class or the relative age (young, adult) and the sex (F: female, M: male). (DOCX) [file pone.0093854.s002.docx]

| **Site** | **Burial** | **Age** | **Sex** | **Age (cal BP)** | **δ^13^C‰** | **δ^15^N‰** | **C%** | **N%** | **Col wt%** | **C:N** |
| --- | --- | --- | --- | --- | --- | --- | --- | --- | --- | --- |
| MRS | 5 | 36 – 50 | F | 5912 - 5464 | -20.7 | +10.4 | 37.7 | 13.4 | 1.4 | 3.3 |
|  | 6A | 11 – 20 | - |  | -20.2 | +11.3 | 35.5 | 12.4 | 1.0 | 3.3 |
|  | 7 | 36 – 50 | M |  | -19.6 | +11.2 | 38.8 | 14.4 | 2.5 | 3.2 |
|  | 7A | 36 – 50 | - |  | -21.2 | +11.3 | 42.7 | 15.3 | 1.3 | 3.3 |
|  | 9A | 36 – 50 | M |  | -20.8 | +11.4 | 36.2 | 12.4 | 2.9 | 3.4 |
|  | 13 | >50 | F |  | -20.7 | +10.2 | 39.8 | 14.3 | 2.2 | 3.2 |
|  | 14A | 36 – 50 | M |  | -20.6 | +10.9 | 38.3 | 13.6 | 1.4 | 3.3 |
|  | 18 | 36 – 50 | - |  | -20.9 | +10.9 | 35.0 | 12.5 | 2.1 | 3.3 |
|  | 25 | 36 – 50 | F | 5289 - 4887 | -21.0 | +10.5 | 39.9 | 14.0 | 3.4 | 3.3 |
|  | 27A | 11 – 20 | - |  | -21.2 | +9.4 | 39.4 | 14.1 | 3.1 | 3.3 |
|  | 27B | 36 – 50 | - |  | -20.6 | +11.0 | 40.1 | 14.4 | 1.9 | 3.3 |
|  | 37A | - | M | 6278 - 6003 | -21.0 | +10.3 | 38.2 | 13.6 | 1.8 | 3.3 |
|  | 35A | >50 | - |  | -21.3 | +10.7 | 39.0 | 14.1 | 2.0 | 3.2 |
|  | 41A | 1 – 10 | - |  | -20.9 | +11.2 | 37.1 | 12.9 | 1.2 | 3.3 |
|  | 42 | 36 – 50 | F |  | -20.8 | +11.1 | 41.8 | 15.1 | 1.5 | 3.2 |
| PCG | 1A | 21 – 35 | F |  | -16.2 | +13.2 | 39.3 | 13.4 | 0.9 | 3.4 |
|  | 5 | 36 – 50 | M |  | -15.6 | +13.3 | 34.0 | 11.5 | 1.7 | 3.4 |
|  | 6 | 21 - 35 | M |  | -15.6 | +13.7 | 37.3 | 12.8 | 0.8 | 3.4 |
|  | 9 | >50 | F |  | -16.7 | +12.5 | 36.9 | 12.4 | 0.3 | 3.5 |
|  | 14 | 36 – 50 | - |  | -15.0 | +13.9 | 38.9 | 13.8 | 1.6 | 3.3 |
|  | 15 | 36 – 50 | F |  | -16.3 | +13.2 | 37.0 | 12.6 | 3.8 | 3.4 |
|  | 17 | 11 – 20 | F |  | -16.5 | +12.9 | 34.5 | 11.4 | 1.1 | 3.5 |
|  | 18 | 11 – 20 | - |  | -15.4 | +14.2 | 40.9 | 14.4 | 1.9 | 3.3 |
|  | 19 | 36 – 50 | - |  | -15.2 | +13.2 | 40.6 | 14.2 | 1.9 | 3.3 |
|  | 32 | 11 – 20 | F |  | -15.3 | +14.3 | 38.7 | 13.7 | 1.2 | 3.3 |
|  | 36 | 21 – 35 | F |  | -15.3 | +13.9 | 34.3 | 11.9 | 6.3 | 3.4 |
|  | 52 | 36 – 50 | F |  | -13.7 | +14.0 | 33.9 | 11.7 | 1.3 | 3.4 |
|  | 56 | 36 – 50 | F |  | -12.9 | +15.6 | 35.0 | 12.6 | 1.2 | 3.2 |
| Jab-II | 1 – T10 | Adult | - |  | -10.9 | +17.6 | 42.5 | 14.9 | 2.7 | 3.3 |
|  | 1B – L6C | 36 – 50 | F |  | -12.2 | +14.3 | 41.8 | 14.8 | 4.4 | 3.3 |
|  | 1D – T10 | 21 – 35 | M |  | -11.6 | +16.7 | 31.4 | 11.3 | 1.2 | 3.3 |
|  | 1-1/2 – T10 | 21 - 35 | - |  | -11.2 | +17.6 | 40.0 | 14.0 | 3.3 | 3.3 |
|  | 2A – L6 | Adult | F |  | -11.8 | +16.9 | 35.3 | 12.5 | 1.4 | 3.3 |
|  | 3B – L6.B3 | Adult | M |  | -11.3 | +18.0 | 37.5 | 13.3 | 2.2 | 3.3 |
|  | 3B2 – L1.05 | 36 – 50 | M |  | -11.1 | +17.6 | 35.3 | 12.4 | 2.6 | 3.3 |
|  | 3C – L6.B3 | 1 – 10 | - |  | -11.1 | +18.3 | 33.9 | 11.8 | 1.4 | 3.3 |
|  | 4A – L6.B4 | 36 – 50 | M |  | -11.6 | +18.2 | 35.7 | 12.8 | 3.4 | 3.3 |
|  | 4B – L1.05 | Adult | - |  | -11.9 | +16.5 | 38.9 | 13.4 | 2.9 | 3.4 |
|  | 8 – L1.05 | Adult | - |  | -11.6 | +16.5 | 41.5 | 14.7 | 4.7 | 3.3 |
|  | 10A – L1.25 | 1 – 10 | - |  | -11.4 | +18.5 | 40.4 | 13.8 | 2.1 | 3.4 |
|  | 10B – L1.25 | 36 – 50 | F |  | -10.8 | +17.4 | 41.1 | 14.2 | 1.1 | 3.4 |
|  | 11 – L1.25 | 36 – 50 | M |  | -10.3 | +18.0 | 40.3 | 14.2 | 2.3 | 3.3 |
|  | 12A – L1.25 | >50 | F |  | -10.3 | +18.4 | 42.4 | 15.2 | 7.7 | 3.2 |
|  | 15 – L1.05 | 36 – 50 | M |  | -11.4 | +16.7 | 30.4 | 10.5 | 2.1 | 3.4 |
|  | 16B – L1.05 | 1 – 10 | - |  | -11.2 | +17.4 | 43.5 | 15.4 | 3.7 | 3.3 |
|  | 17A – L1.05 | 36 – 50 | M |  | -11.1 | +17.5 | 37.5 | 13.4 | 3.8 | 3.3 |
|  | 17C – L2 | Adult | - |  | -18.4 | +11.1 | 34.5 | 12.3 | 2.5 | 3.2 |
|  | 24 – L1.1.20 | Adult | M |  | -10.5 | +17.8 | 40.8 | 15.0 | 1.2 | 3.2 |
|  | 24A – L2 | Adult | - |  | -16.2 | +12.7 | 34.9 | 12.2 | 1.6 | 3.3 |
|  | 25A (FS463) – L2.65 | 36 – 50 | F |  | -11.1 | +18.3 | 39.7 | 14.0 | 4.0 | 3.3 |
|  | 26 – L2.05 | 1 – 10 | - | 2435 - 2144 | -12.0 | +17.6 | 43.0 | 15.2 | 4.9 | 3.3 |
|  | 27A – L2.T15 | Adult | - |  | -10.8 | +18.8 | 40.2 | 14.3 | 3.2 | 3.3 |
|  | 32A – L2.16 | 21 – 35 | - | 2710 - 2362 | -10.7 | +18.7 | 44.3 | 15.5 | 1.8 | 3.3 |
|  | 35A – L2.05 | 1 – 10 | - |  | -10.8 | +18.3 | 40.6 | 14.3 | 4.6 | 3.3 |
|  | 36A – L2.05 | 21 – 35 | M |  | -10.5 | +17.7 | 40.5 | 14.3 | 2.7 | 3.3 |
|  | 36B – L2.05 | 11 – 20 | - |  | -10.8 | +18.0 | 37.1 | 12.8 | 1.9 | 3.4 |
|  | 34 – L2.05 | 21 – 35 | F |  | -10.6 | +18.0 | 40.1 | 14.4 | 5.5 | 3.2 |
|  | 37 – L2.05 | >50 | M |  | -10.8 | +17.9 | 41.7 | 14.9 | 4.1 | 3.3 |
|  | 38 – L2.05 | Adult | - | 2355 - 2145 | -11.5 | +17.7 | 41.0 | 14.2 | 1.9 | 3.4 |
|  | 40 – L2.05 | Adult | F |  | -11.4 | +17.2 | 37.4 | 13.1 | 3.1 | 3.3 |
|  | 41A – L2.05 | Adult | M |  | -10.6 | +17.7 | 41.0 | 14.4 | 2.6 | 3.3 |
|  | 43 – L1.77 | 36 – 50 | M |  | -10.9 | +17.8 | 39.9 | 13.7 | 2.3 | 3.4 |
|  | 102 – L1.75 | Adult | F |  | -15.6 | +13.1 | 42.3 | 15.4 | 2.6 | 3.2 |
|  | 104A – L1.85 | 11 – 20 | - |  | -11.2 | +18.8 | 32.0 | 10.9 | 3.1 | 3.4 |
|  | 106 – L1.90 | 36 - 50 | M |  | -11.2 | +17.5 | 41.8 | 14.7 | 2.8 | 3.3 |
|  | 108 – L2.05 | 36 – 50 | F |  | -10.9 | +17.2 | 41.8 | 15.1 | 4.7 | 3.2 |
|  | 107 – L1.85 | >50 | M |  | -10.6 | +17.8 | 41.1 | 14.7 | 3.8 | 3.3 |
|  | 110 – L2 | 21 – 35 | M |  | -10.5 | +17.7 | 37.7 | 13.5 | 2.9 | 3.3 |
|  | 111/112 – TL1, L2 | 36 – 50 | F |  | -11.3 | +17.5 | 39.5 | 13.9 | 3.1 | 3.3 |
|  | 114 (FS2195) – L6 | >50 | - |  | -11.3 | +18.4 | 41.8 | 15.1 | 7.6 | 3.2 |
|  | 115A – L6 | 1 – 10 | - |  | -12.6 | +20.1 | 43.0 | 15.3 | 9.3 | 3.3 |
|  | 115B (FS2137) – L6 | >50 | M |  | -11.4 | +17.5 | 41.9 | 15.3 | 6.3 | 3.2 |
|  | 118 – L6 | 21 – 35 | - |  | -11.5 | +17.8 | 41.5 | 14.9 | 4.3 | 3.2 |
|  | 120 – L2 | 11 – 20 | - |  | -10.9 | +20.5 | 40.6 | 13.6 | 1.3 | 3.5 |
|  | 121 (FS2138) – L6 | 36 – 50 | M |  | -10.9 | +17.3 | 40.4 | 14.3 | 1.6 | 3.3 |
| G-IV | 1 | - | - | 928 - 763 | -10.3 | +18.3 | 39.7 | 14.4 | 4.1 | 3.2 |
|  | 2 | - | - |  | -11.4 | +17.3 | 31.0 | 10.4 | 0.8 | 3.5 |
|  | 4 | Adult | - |  | -11.1 | +17.4 | 39.3 | 14.2 | 4.4 | 3.2 |
|  | 5 | Adult | - |  | -11.6 | +17.8 | 35.3 | 11.9 | 1.2 | 3.5 |
|  | 6 | Adult | M |  | -10.5 | +17.1 | 42.9 | 15.8 | 6.7 | 3.2 |
|  | 7 | Young | F | 913 - 739 | -14.1 | +16.2 | 42.4 | 15.3 | 3.5 | 3.2 |
|  | 9 | - | - |  | -10.9 | +17.4 | 42.3 | 14.7 | 2.9 | 3.4 |

**Table S2**: **Bone collagen δ^13^C and δ^15^N values of humans. Also show the age class or the relative age (young, adult) and the sex (F: female, M: male).**
