# Supplementary material for: Long-Term Resilience of Late Holocene Coastal Subsistence System in Southeastern South America
Source: PLoS One. 2014 Apr 9;9(4):e93854. doi: 10.1371/journal.pone.0093854 (PMC3981759; doi:10.1371/journal.pone.0093854)
Supplement: Table S3 — One-way ANOVA showing a general lack of significant isotopic differences between sexes and age at MRS, Jab-II and PCG. Data from G-IV was not sufficient to be tested statistically. Because of the limited information about the age, individuals from MRS and PCG were sorted out in two categories: <36 and >36 years old. At Jab-II, individuals belonging to the age class 11–20 years old show higher δ15N values (mean 19.1±1.0%, n = 3) than individuals belonging to the age class 36–50 yrs (mean 17.3±0.9%, n = 13). (DOCX) [file pone.0093854.s003.docx]

| Site | Age | |  | Sex | |  |
| --- | --- | --- | --- | --- | --- | --- |
|  | δ^13^C | δ^15^N | n | δ^13^C | δ^15^N | n |
| MRS | P = 0.9442 | P = 0.5243 | 14 | P = 0.3831 | P = 0.242 | 8 |
| Jab-II | P = 0.2047 | P = 0.0272 | 34 | P = 0.1087 | P = 0.0855 | 27 |
| PCG | P = 0.2874 | P = 0.9511 | 13 | P = 0.7866 | P = 0.7901 | 10 |

**Table S3**: **One-way ANOVA reveals a general lack of significant isotopic differences between sexes and age at MRS, Jab-II and PCG**. Data from G-IV was not sufficient to be tested statistically. Because of the limited information about the age, individuals from MRS and PCG were sorted out in two categories: <36 and >36 years old. At Jab-II, individuals belonging to the age class 11-20 years old show higher δ^15^N values (mean 19.1±1.0‰, n = 3) than individuals belonging to the age class 36-50 yrs old (mean 17.3±0.9‰, n = 13).
