# Supplementary material for: Long-Term Resilience of Late Holocene Coastal Subsistence System in Southeastern South America
Source: PLoS One. 2014 Apr 9;9(4):e93854. doi: 10.1371/journal.pone.0093854 (PMC3981759; doi:10.1371/journal.pone.0093854)
Supplement: Table S4 — Collagen amino acid δ13C values for humans from Jab-II (n = 10) and G-IV (n = 7). (DOCX) [file pone.0093854.s004.docx]

| Site/individuals | | | | | | | | | | | | | | | | |  |  |
| --- | --- | --- | --- | --- | --- | --- | --- | --- | --- | --- | --- | --- | --- | --- | --- | --- | --- | --- |
|  | **Jab-II** | | | | | | | | | | | **G-IV** | | | | | | |
|  | 1B | | 8 | 17C | 24A | 35A | 102 | 107 | 108 | 115B | 118 | 1 | 2 | 4 | 5 | 6 | 7 | 9 |
| δ^13^CAsp | -13.7 | | -12.0 | -19.3 | -17.5 | -11.8 | -17.0 | -11.4 | -12.9 | -13.4 | -14.0 | -12.4 | -12.3 | -13.6 | -13.7 | -12.2 | -14.7 | -12.2 |
| δ^13^CHyp | -13.0 | | -12.4 | -18.5 | -16.4 | -11.2 | -15.8 | -11.6 | -12.1 | -12.5 | -12.8 | -11.7 | -11.6 | -12.0 | -12.2 | -11.4 | -13.8 | -11.4 |
| δ^13^CSer | -6.6 | | -4.3 | -12.8 | -11.6 | -4.1 | -9.5 | -2.8 | -3.4 | -4.2 | -6.4 | -2.2 | -3.3 | -5.1 | -2.9 | -3.0 | -7.9 | -2.4 |
| δ^13^CGlu | -13.7 | | -12.5 | -18.8 | -16.9 | -10.3 | -17.2 | -11.0 | -11.2 | -12.6 | -11.7 | -11.0 | -12.0 | -13.1 | -12.6 | -11.4 | -15.7 | -11.3 |
| δ^13^CHyl | -14.5 | | -13.3 | -19.6 | -17.4 | -13.7 | -17.5 | -13.2 | -13.5 | -13.0 | -12.6 | -13.9 | -11.7 | -12.4 | -13.7 | -13.1 | -14.4 | -12.6 |
| δ^13^CGly | -6.5 | | -4.5 | -14.8 | -12.0 | -5.6 | -10.0 | -3.8 | -4.6 | -5.2 | -5.1 | -2.7 | -4.1 | -4.6 | -3.5 | -3.7 | -8.8 | -3.1 |
| δ^13^CAla | -17.0 | | -14.9 | -20.6 | -19.6 | -14.3 | -18.7 | -13.6 | -14.1 | -15.3 | -15.2 | -14.6 | -15.0 | -15.7 | -14.7 | -14.1 | -20.6 | -13.7 |
| δ^13^CPro | -13.6 | | -12.6 | -18.9 | -17.2 | -11.5 | -16.0 | -12.2 | -12.4 | -12.9 | -12.6 | -12.1 | -11.6 | -12.1 | -12.2 | -11.8 | -14.3 | -11.7 |
| δ^13^CVal | -17.0 | | -17.0 | -24.2 | -22.1 | -15.8 | -21.3 | -16.8 | -16.6 | -16.7 | -17.0 | -16.0 | -16.4 | -16.2 | -16.3 | -15.8 | -20.7 | -15.5 |
| δ^13^CThr | -5.4 | | -5.7 | -12.0 | -12.3 | -6.4 | -10.4 | -7.0 | -6.6 | -8.1 | -8.2 | -4.4 | -8.6 | -8.4 | -7.4 | -5.6 | -6.2 | -7.8 |
| δ^13^CIso | -13.3 | | -13.2 | -19.2 | -18.1 | -11.9 | -17.0 | -11.9 | -12.3 | -13.1 | -12.9 | -12.6 | -11.1 | -11.9 | -11.5 | -11.6 | -15.4 | -12.1 |
| δ^13^CLeu | -19.7 | | -19.7 | -26.4 | -25.0 | -18.7 | -23.7 | -19.1 | -19.7 | -20.0 | -20.0 | -18.8 | -18.5 | -18.7 | -19.4 | -18.4 | -23.5 | -18.7 |
| δ^13^CLys | -14.5 | | -14.2 | -20.7 | -18.7 | -13.4 | -17.6 | -13.7 | -14.4 | -14.4 | -14.2 | -13.7 | -12.6 | -12.8 | -14.1 | -13.6 | -15.5 | -13.3 |
| δ^13^CArg | -15.6 | | -15.0 | -21.5 | -19.9 | -14.0 | -18.7 | -13.9 | -14.7 | -14.8 | -15.0 | -14.5 | -13.9 | -14.5 | -14.3 | -14.3 | -18.4 | -13.9 |
| δ^13^CPhe | -21.2 | | -21.0 | -25.4 | -24.3 | -20.8 | -23.6 | -21.6 | -21.3 | -21.5 | -22.3 | -20.9 | -20.5 | -21.4 | -21.0 | -20.8 | -23.3 | -20.9 |
| δ^13^CBulk | -12.2 | | -11.6 | -18.4 | -16.2 | -10.8 | -15.6 | -10.6 | -10.9 | -11.4 | -11.5 | -10.3 | -11.4 | -11.1 | -11.6 | -10.5 | -14.1 | -10.9 |
| δ^15^NBulk | 14.3 | | 17.8 | 11.1 | 12.7 | 18.3 | 13.1 | 17.8 | 17.4 | 17.5 | 17.8 | 18.3 | 17.3 | 17.4 | 16.5 | 17.1 | 16.2 | 17.2 |

**Table S4**: **Collagen amino acid δ^13^C values for humans from Jab-II (n = 10) and G-IV (n = 7).**
