# Supplementary material for: Long-Term Resilience of Late Holocene Coastal Subsistence System in Southeastern South America
Source: PLoS One. 2014 Apr 9;9(4):e93854. doi: 10.1371/journal.pone.0093854 (PMC3981759; doi:10.1371/journal.pone.0093854)
Supplement: Table S5 — Ceramic sherds selected for lipid analysis by GCMS and GC-c-IRMS. FA (Cx:y) - fatty acids with carbon length x and number of unsaturations y, br -branched chain acids, phy- phytanic acid, TMTD - 4,8,12-trimethyltridecanoic acid. APFA (Cn) - ω-(o-alkylphenyl) alkanoic acids with carbon length n. tr - trace. DCx - α,ω-dicarboxylic acids with carbon length x. P - interior, E - exterior. Aquatic oils are interpreted from the presence of isomers of APFA (C20 or C22) and at least one isoprenoid fatty acids (pri, phy or TMTD). Resins are interpreted from the presence of triterpenes. Plant oils are interpreted from the presence of long chain fatty acids, dicarboxylique acids and the presence of isomers of C18 APFA. A high abundance of C12∶0 could be consistent with Palm Kernel. Aquatic (marine) fats are defined on the isotopic characteristics of the C16 and C18 saturated fatty acids. (DOCX) [file pone.0093854.s005.docx]

**Table S5.** Ceramic sherds selected for lipid analysis by GCMS and GC-c-IRMS**.** FA (Cx:y) - fatty acids with carbon length x and number of unsaturations y, br -branched chain acids, phy- phytanic acid, TMTD - 4,8,12-trimethyltridecanoic acid. APFA (Cn) - ω-(o-alkylphenyl) alkanoic acids with carbon length n. tr - trace. DCx - α,ω-dicarboxylic acids with carbon length x. P - interior, E - exterior.

Aquatic oils are interpreted from the presence of isomers of APFA (C_20_ or C_22_) and at least one isoprenoid fatty acids (pri, phy or TMTD). Resins are interpreted from the presence of triterpenes. Plant oils are interpreted from the presence of long chain fatty acids, dicarboxylique acids and the presence of isomers of C18 APFA. A high abundance of C_12:0_ could be consistent with Palm Kernel. Aquatic (marine) fats are defined on the isotopic characteristics of the C_16_ and C_18_ saturated fatty acids.

| Laboratory code | Lipid conc. *(µg mg^-1^)* | Compound detected | C_16:0_ δ^13^C‰ | C_18:0_ δ^13^C‰ | Interpretation |
| --- | --- | --- | --- | --- | --- |
| G16P | 2.94 | FA (C_14:0-22:0,_ C_18:1-22:1_, C_15-17br_), DC (C_9-12_), APFA (C_18-20 tr_) phy, TMTD, triterpenes | -24.3 | -23.3 | Marine, resins |
| G17P | 0.02 | FA (C_16:0-18:0_), DC (C_9_) | -26.8 | -28.5 |  |
| G18P | 0.10 | FA (C_14:0-24:0,_ C_16:1-22:1_, C_15-17br_), DC ( C_9-13_), phy, TMTD | -23.1 | -24.7 | Marine |
| G18E | 0.39 | FA (C_14:0-24:0,_ C_18:1-22:1_, C_15-17br_), DC (C_9-13_), APFA (C_16-22_), phy, pri, TMTD | -22.8 | -24.2 | Marine |
| G19P | 0.02 | FA (C_16:0-18:0,_ C_18:1 (tr)_) | -26.1 | -27.9 |  |
| G20P | 0.21 | FA (C_14:0-18:0_, C_18:1 (tr)_) | -23.6 | -25.5 | Marine |
| G21P | 0.05 | FA (C_14:0-24:0_, C_18:1-22:1_, C_15-17br_), DC (C_9-11_), phy | -23.7 | -23.7 | Marine |
| G22P | 0.20 | FA (C_12:0-C28:0,_ C_18:1-20:1_, C_15-17br_), DC (C_9-11, 12-24 (tr)_), APFA(C_18_), phy | -26.5 | -25.7 | Plant (Palm kernel?) |
| G23P | tr | FA (C_16:0-18:0_) |  |  |  |
| G24P | 0.08 | FA (C_14:0-28:0_, C_18:1_), DC (C_22-24(tr)_), triterpenes | -26.4 | -26.7 | Resins |
| G25P | 0.01 | FA (C_16:0-18:0,_ C_18:1 (tr)_) |  |  |  |
| G26P | 0.25 | FA (C_14:0-18:0_, C_18:1_), ), DC (C_9-13_), phy, TMTD | -21.5 | -20.6 | Marine |
| G26E | 0.52 | FA (C_14:0-18:0_, C_16:1-18:1_), DC (C_9-13_), phy, TMTD | -21.8 | -21.5 | Marine |
| G27P | tr | FA (C_16:0-18:0_) |  |  |  |
