# Supplementary material for: Long-Term Resilience of Late Holocene Coastal Subsistence System in Southeastern South America
Source: PLoS One. 2014 Apr 9;9(4):e93854. doi: 10.1371/journal.pone.0093854 (PMC3981759; doi:10.1371/journal.pone.0093854)
Supplement: Table S6 — Bulk isotope characteristics of charred deposits from the interior of potsherds from G-IV. (DOCX) [file pone.0093854.s006.docx]

**Table S6**: Bulk isotope characteristics of charred deposits from the interior of potsherds from G-IV.

| **Laboratory code** | **%C** | **δ^13^C‰** | **%N** | **δ^15^N‰** | **C:N** |
| --- | --- | --- | --- | --- | --- |
| G16 | 24.9 | -22.6 | 2.8 | 8.7 | 10.2 |
| G17 | 8.24 | -24.5 | 0.8 | 12.7 | 12.5 |
| G18 | 26.9 | -25.5 | 3.1 | 7.9 | 10.0 |
| G20 | 32.9 | -23.9 | 2.8 | 8.6 | 13.5 |
| G21 | 10.9 | -24.3 | 1.2 | 8.4 | 10.8 |
| G26 | 37.1 | -25.8 | 2.6 | 6.7 | 16.3 |
| G28 | 4.0 | -23.2 | 0.3 | 17.2 | 17.6 |
